# NMDARs activation regulates endothelial ferroptosis via the PP2A-AMPK-HMGB1 axis

**DOI:** 10.1038/s41420-023-01794-3

**Published:** 2024-01-17

**Authors:** Wei-Min Han, Yi-Xiang Hong, Guo-Sheng Xiao, Rui-Ying Wang, Gang Li

**Affiliations:** 1 Xiamen Cardiovascular Hospital of Xiamen University, School of Medicine, Xiamen University, Xiamen, Fujian 361008 China; 2 Xiamen Key Laboratory of Cardiovascular Disease, Xiamen, Fujian 361008 China

**Keywords:** Cell death, Drug development

## Abstract

N-methyl-D-aspartate receptors (NMDARs) are ligand-gated, voltage-dependent channels of the ionotropic glutamate receptor family. The present study explored whether NMDAR activation induced ferroptosis in vascular endothelial cells and its complicated mechanisms in vivo and in vitro. Various detection approaches were used to determine the ferroptosis-related cellular iron content, lipid reactive oxygen species (LOS), siRNA molecules, RNA-sequence, MDA, GSH, and western blotting. The AMPK activator Acadesine (AICAR), HMGB1 inhibitor glycyrrhizin (GLY), PP2A inhibitor LB-100, and NMDAR inhibitor MK801 were used to investigate the involved in vivo and in vitro pathways. The activation of NMDAR with L-glutamic acid (GLU) or NMDA significantly promoted cellular ferroptosis, iron content, MDA, and the PTGS2 expression, while decreasing GPX4 expression and GSH concentration in human umbilical vein endothelial cells (HUVECs), which was reversed by ferroptosis inhibitors Ferrostatin-1(Fer-1), Liproxstatin-1 (Lip-1), or Deferoxamine (DFO). RNA-seq revealed that ferroptosis and SLC7A11 participate in NMDA or GLU-mediated NMDAR activation. The PP2A-AMPK-HMGB1 pathway was majorly associated with NMDAR activation-induced ferroptosis, validated using the PP2A inhibitor LB-100, AMPK activator AICAR, or HMGB1 siRNA. The role of NMDAR in ferroptosis was validated in HUVECs induced with the ferroptosis activator errasin or RSL3 and counteracted by the NMDAR inhibitor MK-801. The in vivo results showed that NMDA- or GLU-induced ferroptosis and LOS production was reversed by MK-801, LB-100, AICAR, MK-801, and GLY, confirming that the PP2A-AMPK-HMGB1 pathway is involved in NMDAR activation-induced vascular endothelium ferroptosis. In conclusion, the present study demonstrated a novel role of NMDAR in endothelial cell injury by regulating ferroptosis via the PP2A-AMPK-HMGB1 pathway.

## Introduction

Vascular endothelial cells (VECs), located in an oxygen-rich vascular environment, are vital for supporting the structure and biological function of blood vessels and maintaining vascular tension by mediating the balance of vasodilation, contraction, growth inhibition, and growth promotion [1] as well as anti-inflammatory or pro-inflammatory effects [2, 3]. VEC dysfunction may result in arteriosclerosis [4, 5] and cardiovascular diseases such as thromboangiitis obliterans [6, 7], which are closely related to cardiovascular risk factors. In contrast to atherosclerosis, endothelial dysfunction can also be reversed. Therefore, early prevention and treatment should be performed to prevent vascular disease [8–11]. Ferroptosis is a new type of regulated cell death [12, 13] and was first reported in 2012 by Dixon et al. It is an iron-dependent lipid peroxidation and is biochemically distinct from other forms of cell death, such as necrosis [14], apoptosis, and pyroptosis [15–17]. It was found that increasing cellular ferroptosis further aggravated functional damage to many diseases, such as ischemic diseases, tumors, and tissue injuries, including traumatic brain injury, neurodegenerative disease [18], folic acid-induced kidney injury, and CAD [19–21]. However, the roles of ferroptosis in endothelial cell function and vascular dysfunction have not been explored under NMDAR-activated conditions.

N-methyl-D-aspartate receptors (NMDARs) are ligand-gated voltage-dependent channels that belong to the ionotropic glutamate receptor family and are composed of seven subunits (GluN1, GluN2A–D, and GluN3A/B), located at cell-cell contact sites, particularly for excitatory neuronal synaptic communication in the central nervous system [22–25]. In this study, we reveal a novel perspective on VEC pathogenesis and propose an effective targeted treatment strategy for vascular diseases by inhibiting NMDAR activation-mediated endothelial cell ferroptosis by targeting the PP2A-AMPK-HMGB1 axis, which is clinically promising in preventing VEC dysfunction and vascular degeneration in patients with early stage AS, thrombosis, and infection.

## Results

### NMDAR activation induces ferroptosis in VECs following treatment with NMDA or GLU

**Fig. 1:**
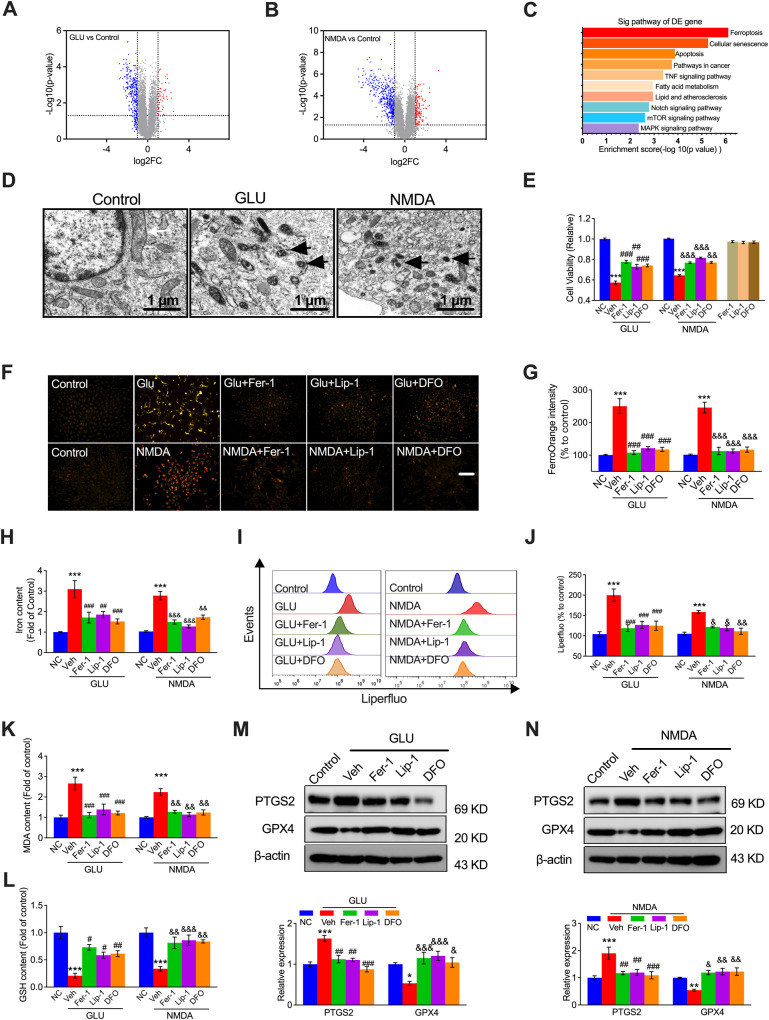
Ferroptosis participates in NMDAR activation-mediated endothelial cell damage in vitro. RNA sequencing was performed after in HUVECs treated with GLU (20 mM) or NMDA (1 mM) for 24 h, and the results of volcano plot (**A**, **B**) and KEGG analysis (**C**) were shown. **D** Ultrastructure of the mitochondria in HUVECs treated with GLU or NMDA, visualized by TEM. Black arrowheads: shrunken mitochondria (Scale bars, 1 μm). **E** MTT assay and (**F**) ferrous ion levels visualized by FerroOrange staining (Scale bars, 200 μm) and (**G**) corresponding quantification showing cell viability and ferrous ion levels in HUVECs pretreated with ferroptosis inhibitors (Fer-1: 10 μM, Lip-1: 2 μM, or DFO: 10 μM) for 1 h followed by GLU and NMDA treatment for 24 h. The relative levels of iron content (**H**), flow cytometry plots (**I**), and quantification of lipid ROS (LOS) production (**J**), MDA (**K**), and GSH (**L**) in HUVECs pretreated with Fer-1 (10 μM), Lip-1 (2 μM), or DFO (10 μM) for 1 h followed by GLU or NMDA treatment for 24 h. The relative levels of ferroptosis biomarkers PTGS2 and GPX4 shown by immunoprotein blots and quantification in the presence of GLU (**M**) or NMDA (**N**) and treatment with Fer-1 (10 μM), Lip-1 (2 μM), or DFO (10 μM). ^*^P < 0.05, ^**^P < 0.01 and ^***^P < 0.001 *vs* control; ^#^*P* < 0.05, ^##^*P* < 0.01 and ^###^*P* < 0.001 vs GLU; ^&^*P* < 0.05, ^&&^*P* < 0.01 and ^&&&^*P* < 0.001 *vs* NMDA.

To determine endothelial cell fate and provide insight into the underlying mechanisms of VECs under NMDAR activation, NMDA and GLU were used to induce VEC abnormalities to establish the NMDAR activation model. RNA sequencing was performed to determine the pathways involved in the NMDAR-activated HUVECs. After HUVECs were treated with NMDA or GLU, significantly downregulated and upregulated genes were observed in the volcano plot and compared with the control cells (Fig. 1A, B). Kyoto Encyclopedia of Genes and Genomes (KEGG) enrichment analyses of the differentially expressed genes showed that ferroptosis was the top pathway (Fig. 1C). We then used transmission electron microscopy (TEM) to determine whether ferroptosis was involved in NMDA- or GLU-treated HUVECs. The cells exhibited distinctive morphological features with smaller mitochondria and increased membrane density (Fig. 1D), similar to those observed in ferroptosis-induced injury. To further investigate whether ferroptosis is involved in NMDA- or GLU-treated HUVECs, we used three ferroptosis inhibitors, Fer-1, Lip-1, and DFO. As shown in the cell viability analysis by MTT assay, a significant rescue effect on cell viability was observed in the presence of Fer-1, Lip-1, and DFO in NMDA- or GLU-induced HUVECs (Fig. 1E). Since cellular iron accumulation is a typical hallmark of ferroptosis, to further validate that NMDAR activation can induce ferroptosis in HUVECs, we used a ferrous ion dye (FerroOrange) and an iron content assay kit. FerroOrange intensity staining (Fig. 1F, G) and iron content assay (Fig. 1H) showed that NMDA- or GLU-treated cells accumulated excessive ferrous ions, which were significantly blocked by Fer-1, Lip-1, or DFO. The accumulation of ferrous ions can increase oxidative stress, including LOS. Subsequently, LOS levels were determined by flow cytometry using the fluorescent probe Liperfluo. NMDA or GLU treatment led to increase in the cellular LOS level, which can be suppressed by pretreatment with the ferroptosis inhibitors Fer-1, Lip-1, or DFO (Fig. 1I, J). To further investigate NMDAR activation on oxidative stress-related VEC fate, we measured the levels of MDA, the most prevalent byproduct of LOS. We found that MDA levels were significantly elevated in NMDA- or GLU-treated HUVECs, which could be suppressed by Fer-1, Lip-1, or DFO (Fig. 1K). The tripeptide GSH serves as a critical protection for cellular antioxidant defense, protecting the structure and function of the cell membrane against peroxides [26]. As shown in Fig. 1L, decreased GSH levels were observed in response to NMDA- and GLU treatment in HUVECs, indicating that NMDAR activation significantly disrupts the cellular antioxidant capacity of endothelial cells, which can be substantially attenuated by Fer-1, Lip-1, and DFO. To further verify NMDAR activation-induced ferroptosis in HUVECs, the levels of ferroptosis markers glutathione peroxidase 4 (GPX4) and prostaglandin-endoperoxide synthase 2 (PTGS2) were analyzed by western blotting. Compared with untreated HUVECs, NMDA- and GLU-treated endothelial cells showed increased PTGS2 protein levels and decreased GPX4 levels (Fig. 1M, N), which can be reversed by Fer-1, Lip-1, or DFO treatment, further emphasizing that ferroptosis plays an essential role in oxidative stress-mediated endothelial cell death. These results indicated that ferroptosis is a crucial pathological process in NMDAR activation-induced oxidative stress and VEC death.

### Excessive activation of NMDAR-induced ferroptosis is regulated by the PP2A-AMPK-HMGB1 hierarchy

**Fig. 2:**
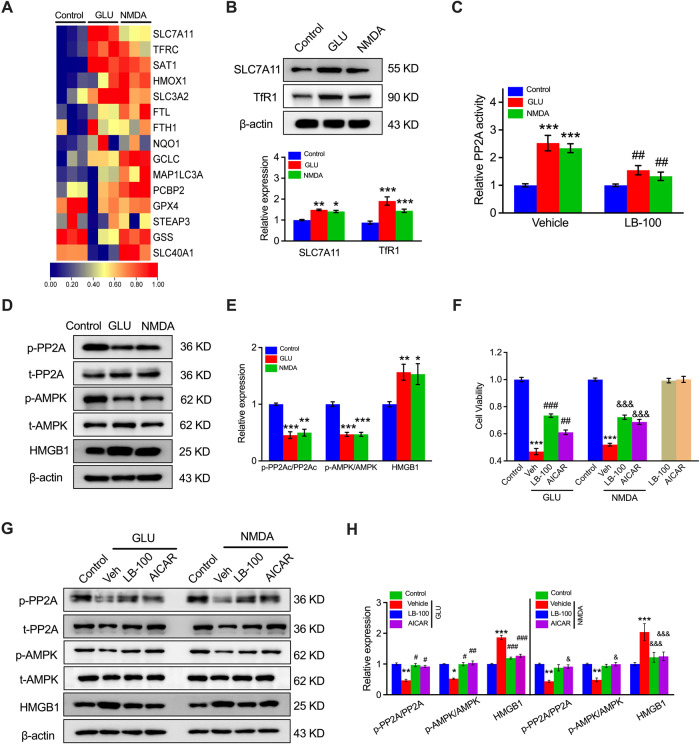
Activation of SLC7A11 via the PP2A-AMPK axis contributes to NMDAR-activated ferroptosis in HUVECs. **A** Differentially expressed genes closely associated with HUVEC ferroptosis in NMDA- or GLU-treated cells determined by RNA-seq. Blue: low expression levels; Red: high expression levels. **B**Protein blotting and quantitation of *SLC7A11* and *TfR1* expression in HUVECs in response to GLU or NMDA treatment. ^*^P < 0.05, ^**^P < 0.01 and ^***^P < 0.001 vs control^.^ **C** The relative PP2A activity of HUVECs after pretreatment with LB-100 (0.5 μM) followed by NMDA or GLU treatment (*n* = 6–8). ^***^P < 0.001 vs control with vehicle; ^##^*P* < 0.01 < 0.001 vs control with LB-100. **D** *P*rotein expression levels and (**E**) quantitation of SLC7A11 upstream, including PP2A, AMPK, and HMGB1, were analyzed by western blotting (*n* = 6). ^*^P < 0.05 ^**^P < 0.01, and ^***^P < 0.001 vs control. **F** MTT assay detected the cell viability of HUVECs treated with NMDA or GLU and/or LB-100 (0.5 μM) and AICAR (25 μM) for 24 h (*n* = 6). ^***^P < 0.001 *vs* control; ^##^*P* < 0.01 and ^###^*P* < 0.001 *vs* GLU; ^&&&^*P* < 0.001 *vs* NMDA. **G** Protein expression levels and (**H**) quantitation of LB-100 and AICAR with the relative expression of p-PP2A, P*P*2A, p-AMPK, p-AMPK, and HMGB1 in HUVECs (*n* = 5). ^*^P < 0.05, ^**^P < 0.01 and ^***^P < 0.001 vs control; ^#^*P* < 0.05, ^##^*P* < 0.01, and ^###^*P* < 0.001 vs GLU; ^&^*P* < 0.05 and ^&&&^*P* < 0.001 vs NMDA.

Further analyses were performed on the RNA sequence of NMDA- or GLU-treated HUVECs. Multiple genes closely associated with ferroptosis were also analyzed. We found that solute carrier family 7 member 11 (*SLC7A11*) and transferrin receptor (*TFRC*, also known as *TfR1*) were the most dramatically upregulated genes in NMDA- and GLU-treated HUVECs (Fig. 2A), as validated by western blot analysis (Fig. 2B). Several studies have shown that SLC7 is a critical upstream regulator of ferroptosis [27–30]. Recently, a report documented that *SLC7A11* expression is mediated by the PP2A-AMPK pathway [31] and the transcription factor HMGB1. We then explored PP2A activity (Fig. 2C), phosphorylation of PP2A and AMPK, and expression of HMGB1 (Fig. 2D, E). PP2A activity was elevated (Fig. 2C), PP2A and AMPK phosphorylation were decreased (Fig. 2D), and the expression of HMGB1 was elevated after GLU or NMDA treatment (Fig. 2D). To confirm that the PP2A-AMPK pathway participates in NMDAR-mediated ferroptosis, cell viability was determined by MTT assay using the PP2A blocker LB-100 and AMPK activator AICAR in the presence or absence of NMDA and GLU. Significant rescue effects of LB-100 and AICAR were observed in NMDA- and GLU-treated cells (Fig. 2F). Moreover, GLU- or NMDA-regulated PP2A and AMPK phosphorylation and HMGB1 expression were reversed by LB-100 or AICAR pre-treatment (Fig. 2G, H). These results suggest that the PP2A-AMPK pathway is involved in NMDAR activation by regulating HMGB1 expression.

### HMGB1 participates in NMDAR activation-induced HUVEC ferroptosis

**Fig. 3:**
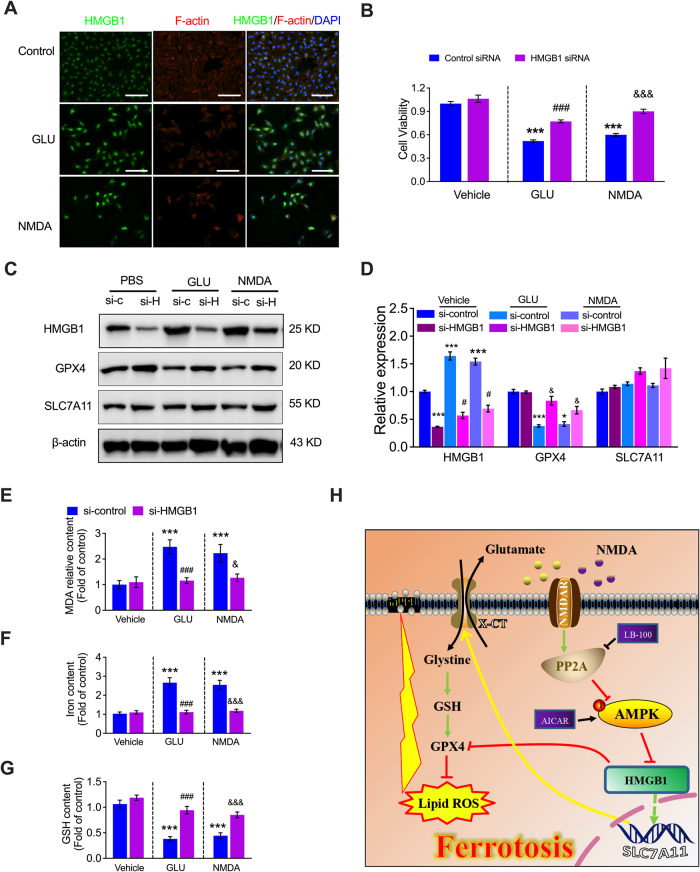
Knockdown of HMGB1 suppresses NMDAR activation-induced ferroptosis. **A** Representative image of immunostaining of HMGB1 in the presence of GLU or NMDA in HUVECs. **B** Representative cell viability of the control siRNA or HMGB1 siRNA-transfected HUVECs in the presence of GLU or NMDA with MTT assay (*n* = 5). **C** Protein expression levels and (**D**) quantitation of HMGB1, GPX4, and SLC7A1 in the control siRNA or HMGB1 siRNA-transfected HUVECs in the presence of GLU or NMDA (*n* = 5). **E** MDA content, (**F**) iron content, and (**G**) GSH levels in the control siRNA or HMGB1 siRNA-transfected HUVECs in the presence of GLU or NMDA (*n* = 5). **H** Scheme of the mechanism of NMDAR inhibitor MK-801 in VEC ferroptosis. ^*^P < 0.05, ^***^P < 0.001 *vs* control siRNA; ^###^*P* < 0.001 vs control siRNA + GLU, ^&^*P* < 0.05 and ^&&&^*P* < 0.001 vs control siRNA + NMDA.

Interestingly, HMGB1 levels were significantly elevated in HUVECs in the presence of GLU or NMDA (Fig. 3A). To further validate whether HMGB1 is involved in NMDAR activation-regulated ferroptosis, we used HMGB1 siRNA to investigate the role of HMGB1 after NMDAR activation. Following knockdown of HMGB1 with siRNA, GLU- or NMDA-induced cell death was dramatically suppressed (Fig. 3B). Interestingly, the GLU- or NMDA-promoted ferroptosis-related protein GPX4 was significantly increased after HMGB1 knockdown (Fig. 3C, D). However, there was no significant change in GLU- or NMDA-promoted SLC7A11 expression due to siHMGB1 (Fig. 3C, D). Interestingly, GLU- or NMDA-regulated MDA, iron content, and GSH content in HUVECs was also reversed by f HMGB1 knockdown (Fig. 3E–G). These results suggest that HMGB1 is a critical regulator of ferroptosis that is regulated by NMDAR activation.

### MK-801-mediated inhibition of NMDAR suppresses Erastin- and RSL3-induced ferroptosis

**Fig. 4:**
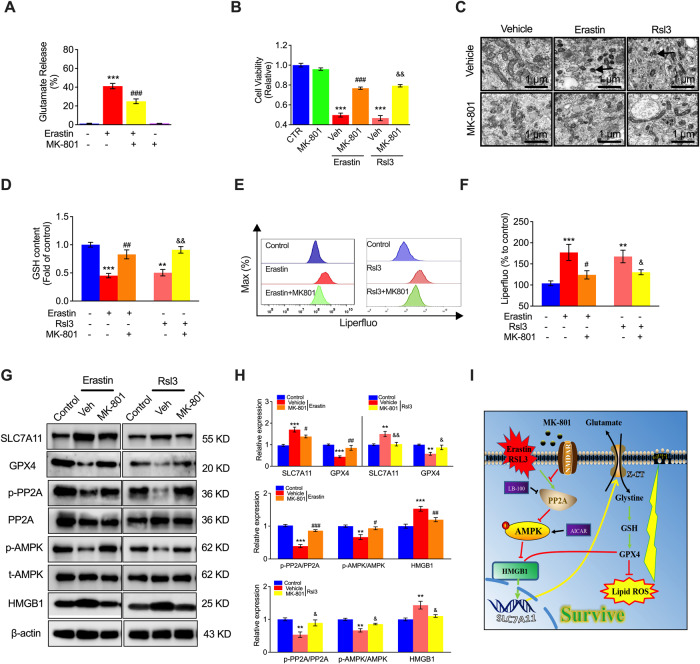
Inhibition of NMDAR ameliorates Erastin- or RSL3-induced ferroptosis in vitro. **A** Glutamate release in HUVECs treated with Erastin in the presence of the NMDAR inhibitor MK801. **B** MTT assay showing HUVECs treated with Erastin or RSL3 in the presence of MK-801. **C** Ultrastructure of mitochondria in HUVECs treated with Erastin or RSL3 in the presence of MK-801. Black arrowheads: shrunken mitochondria. Scale bars, 5 μm. **D** GSH content in HUVECs pretreated with ferroptosis inducers (Erastin, 10 μM; RSL3, μM) for 24 h and then treated with MK801. **E** Representative flow cytometry image and quantification (**F**) of LOS production in HUVECs pretreated with a ferroptosis inducer (Erastin, 10 μM; RSL3, μM) for 24 h and treated with MK801. **G** Immunoblots and quantification (**H**) of SLC7A11, GPX4, p-PP2A, PP2A, p-AMPK, AMPK, and HMGB1 in HUVECs pretreated with a ferroptosis inducer (Erastin, 10 μM; RSL3, μM) for 24 h and treated with MK801. **I** Scheme of the protective mechanism of inhibiting HMGB1 overexpression by MK-801 treatment against ferroptosis in VECs. ^**^P < 0.01 and ^***^P < 0.001 vs control; ^#^*P* < 0.05, ^##^*P* < 0.01 and ^###^*P* < 0.001 vs Erastin; ^&^*P* < 0.05 and ^&&^*P* < 0.01 vs RSL3.

To confirm that NMDARs are vital in regulating ferroptosis, the NMDAR selective inhibitor MK-801 was used to explore the role of NMDAR after Erastin- and RSL3-induced activation of ferroptosis. We first detected the GLU content in the presence of Erastin with or without MK801. We found that Erastin significantly promoted GLU release in HUVECs, which was inhibited by MK-801 (Fig. 4A), indicating that GLU release is associated with ferroptosis. Moreover, Erastin- and RSL3-induced cell death was significantly inhibited by MK-801 (Fig. 4B). Similar to the results shown in Fig. 1D captured by TEM, Erastin- or RSL3-treated HUVECs exhibited similar morphological, biochemical, and other similarities with NMDA- or GLU-induced ferroptosis, which were ameliorated by MK-801 in HUVECs (Fig. 4C). In addition, Erastin and RSL3 decreased the GSH content in HUVECs, which was reversed by MK-801 treatment (Fig. 4D). Moreover, Erastin- and RSL3-induced LOS production was suppressed by MK801 treatment (Fig. 4E, F). The mechanism by which MK-801 reversed Erastin- and RSL3-altered expression of GPX4, SLC7A11, p-PP2A, p-AMPK, and HMGB1 (Fig. 4G, H) illustrated the mechanism by which NMDAR participated in Erastin- and RSL3-induced VEC ferroptosis via regulation of the PP2A-AMPK-HMGB1 pathway (Fig. 4I).

### NMDAR activation mediates ferroptosis in VECs in vivo

**Fig. 5:**
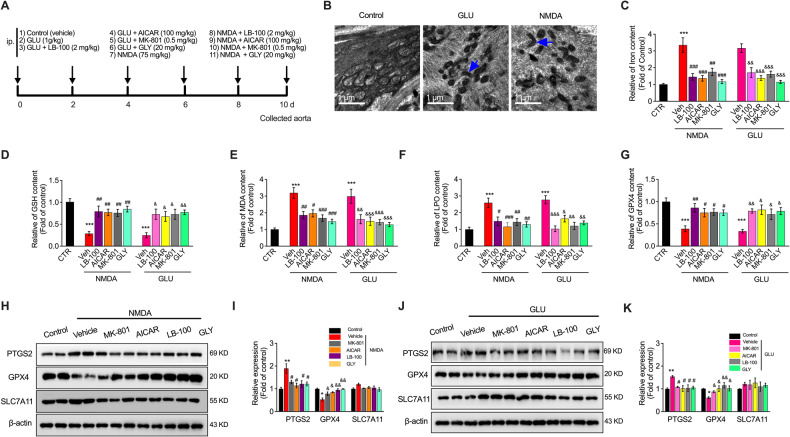
NMDAR and PP2A-AMPK-HMGB1 pathway mediate VEC ferroptosis in vivo. **A** Experimental design summarizing the procedure. **B** Ultrastructure of the mitochondria of the vascular tissue of mice treated with GLU (1 g/kg) or NMDA (75 mg/kg) for 10 days, visualized by TEM. Black arrowheads: shrunken mitochondria. Scale bars, 5 μm. The relative levels of (**C**) iron, (**D**) GSH, (**E**) MDA, (**F**) LPO, and (**G**) GPX4 content in the vascular tissue of the treated mice. **H** Western blot image and (**I**) quantification of PTGS2, GPX4, and SLC7A11 in NMDA and/or LB100-, AICAR-, MK-801-, and GLY-treated mice. ^***^P < 0.001 vs control; ^#^*P* < 0.05, ^##^*P* < 0.01 and ^###^*P* < 0.001 *vs* NMDA, ^&^*P* < 0.05, ^&&^*P* < 0.01 and ^&&&^*P* < 0.001 vs GLU. **J** Western blot image and (**K**) quantification of *P*TGS2, GPX4, and SLC7A11 in GLU and/or LB100-, AICAR-, MK-801-, and GLY-treated mice. ^*^P < 0.05, ^**^P < 0.01 and ^***^P < 0.001 vs control; ^#^*P* < 0.05, ^##^*P* < 0.01 and ^###^*P* < 0.001 vs NMDA/GLU in PTGS2 expression; ^&^*P* < 0.05, ^&&^*P* < 0.01 and ^&&&^*P* < 0.001 vs NMDA/GLU in GPX4 expression.

We investigated the effect of the PP2A blocker LB-100, AMPK activator AICAR, NMDAR inhibitor MK-801, and HMGB1 inhibitor glycyrrhizin (GLY) in vivo in GLU- or NMDA-treated mice (Fig. 5A). Similar to the in vitro results, TEM detection showed that compared to the normal control group, the mitochondrial morphology in the aorta of NMDA- or GLU-treated mice showed a characteristic change in ferroptosis, with the presence of smaller mitochondria and reduced cristae (Fig. 5B). The iron content in the aorta was also significantly elevated by NMDA or GLU treatment, which could be reversed considerably by LB-100, AICAR, MK-801, and GLY treatment (Fig. 5C). In addition, after NMDAR activation, GSH (Fig. 5D) levels decreased, while lipid peroxide MDA (Fig. 5E) and LPO (Fig. 5F) increased in the aortas after GLU or NMDA induction. However, LB-100, AICAR, MK-801, and GLY effectively attenuated this damage. Moreover, GLU- and NMDA-induced GPX4 content and expression decreased, and PTGS2 promotion was significantly suppressed by treatment with MK-801, AICAR, LB-100, and GLY. Besides, the treatment of MK-801, AICAR, LB-100, and GLY had the tendency to reduce the expression of SLC7A11 without significant difference. (Fig. 5G–K).

### NMDAR activation mediates ferroptosis in VECs via the PP2A-AMPK-HMGB1 pathway in vivo

**Fig. 6:**
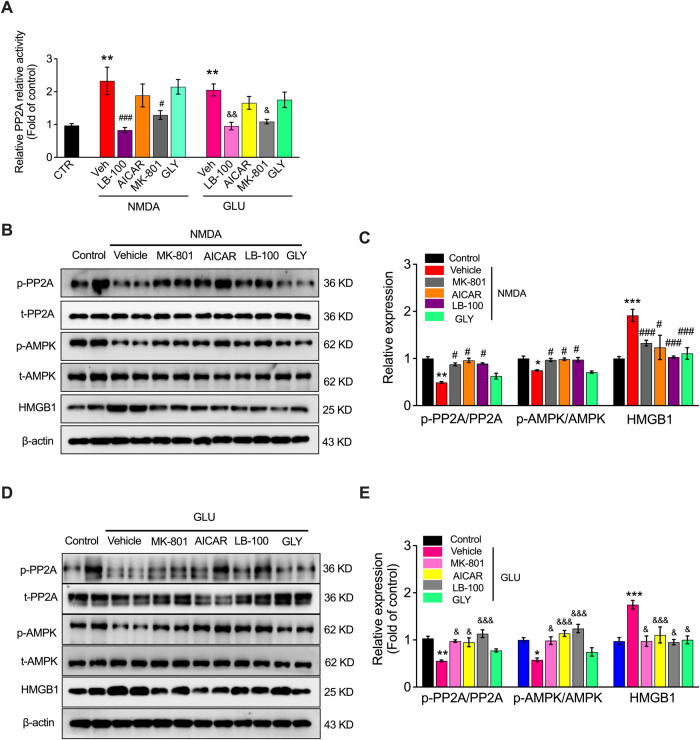
NMDAR activation-mediated VEC ferroptosis is regulated by the PP2A-AMPK-HMGB1 pathway in vivo. **A** PP2A activity in vascular tissue after treatment with LB100, AICAR, MK-801, and GLY in the presence or absence of NMDA or GLU in vivo. **B** Western blot image and (**C**) quantification of p-PP2A, PP2A, p-AMPK, AMPK, and HMGB1 in GLU and/or LB100-, AICAR-, MK-801-, and GLY-treated mice. **D** Western blot image and (**E**) quantification of p-PP2A, PP2A, p-AMPK, AMPK, and HMGB1 in GLU and/or LB100-, AICAR-, MK-801-, and GLY-treated mice. ^*^P < 0.05, ^**^P < 0.01 and ^***^P < 0.001 *vs* control; ^#^*P* < 0.05 and ^###^*P* < 0.001 *vs* NMDA; ^&^*P* < 0.05, ^&&^*P* < 0.01 and ^&&&^*P* < 0.001 vs GLU.

To explore whether the PP2A-AMPK-HMGB1 pathway participates in NMDAR activation-induced vascular damage, we investigated PP2A activity and phosphorylation as well as p-AMPK and HMGB1 expression in the aortas of mice treated with GLU or NMDA in the presence or absence of MK-801, LB-100, AICAR, and GLY. The PP2A content in vascular tissues was promoted by the activation of NMDAR with GLU or NMDA, which was blocked by the LB-100 and MK-801 but not by AICAR or GLY (Fig. 6A), indicating that NMDAR activation-induced PP2A activity was not regulated upstream by AMPK and HMGB1. Furthermore, we found that NMDAR activation downregulated p-PP2A and p-AMPK, upregulated the expression of HMGB1, and restricted NMDAR (MK-801) and PP2A (LB-100); activation of AMPK (AICAR) reversed these effects in vivo (Fig. 6B–E). However, the results showed that inhibition of HMGB1 with GLY did not affect the phosphorylation of PP2A or AMPK, indicating that HMGB1 is downstream of PP2A and AMPK (Fig. 6B–E). These results suggest that NMDAR activation mediates ferroptosis in VECs via the PP2A-AMPK-HMGB1 pathway in ferroptotic mice, and inhibition of NMDAR confers vascular protection against ferroptosis by reversing the downregulation of p-PP2A and p-AMPK, along with the upregulation of HMGB1.

## Discussion

In the present study, we found that activation of NMDAR with L-glutamic acid (GLU) or NMDA promoted ferroptosis by increasing iron content, MDA and the expression of PTGS2, and decreasing the expression of GPX4, which was reversed by ferroptosis inhibitors Fer-1, Lip-1, or DFO. The PP2A-AMPK-HMGB1 pathway was the major pathway associated with NMDAR activation-induced ferroptosis, which was validated using the PP2A inhibitor LB-100, AMPK activator AICAR, or HMGB1 siRNA. The role of NMDAR in ferroptosis was validated by ferroptosis activators Erastin or RSL3, which were counteracted by the NMDAR inhibitor MK-801. The in vivo results showed that NMDA- or GLU-induced ferroptosis was reversed by MK-801, LB-100, AICAR, MK-801, and GLY, confirming that the PP2A-AMPK-HMGB1 pathway is involved in NMDAR activation-induced vascular endothelium ferroptosis. Our study demonstrated a novel role of NMDAR in endothelial cell injury by regulating ferroptosis via the PP2A-AMPK-HMGB1 pathway.

NMDARs play a critical pathological role in chronic peripheral disorders [23, 32–34], such as type 2 diabetes mellitus [35] and cancer [34, 36]. However, the role of the glutamate-NMDAR axis in vascular endothelial cell injury has not been investigated. It was initially reported that non-transferrin-bound iron (NTBI) promotes iron influx into cells and increases the activation of NMDAR, increasing ROS levels and sensitizing neurons to iron death [37]. Thus, we hypothesized that dysregulation of glutamatergic communication via NMDAR between VECs may be involved in vascular injury induced by ferroptosis observed in endothelial cells and may be targeted pharmacologically. As an emerging form of iron-dependent cell death, ferroptosis is characterized by intracellular iron overload and lipid peroxidation [38]. In the present study, we present a novel mechanism of NMDAR activation in the regulation of ferroptosis in VECs. As a new form of endothelial cell death, ferroptosis has the characteristic that the mitochondria in the cells shrink and mitochondrial membrane density increases [39]. Our results showed that after NMDAR activation by treatment with NMDA or GLU in HUVECs, the mitochondria exhibited distinctive morphological features such as smaller size with increased membrane density, similar to the characteristics of ferroptosis. A previous study showed that ferroptosis is a regulated form of cell death attributed to abundant cellular iron levels that imbalance the production and clearance of lipid peroxides [40]. Our results showed a similar effect of ferroptosis by NMDA or GLU treatment on cell viability, FerroOrange intensity, and iron content in HUVECs, which was rescued by the ferroptosis inhibitors Fer-1, Lip-1, and DFO. Ferroptosis is biochemically characterized by the accumulation of lipid peroxides and reactive oxygen species (ROS) [41, 42] and can directly or indirectly inhibit GPX4 [43], which leads to intracellular antioxidant system damage and ROS accumulation in the mitochondria, thereby causing cellular dysfunction. PTGS2, also known as cyclooxygenase 2 (COX2), acts as a dioxygenase and peroxidase, and participates in ferroptosis. LOS production, MDA, and GPX4 and PTGS2 expression in NMDA- or GLU-treated cells and mice changed in our present study and were reversed by a ferroptosis inhibitor, NMDAR inhibitor, HMGB1 inhibitor, or AMPK activator. Moreover, the accumulation of intracellular Fe^2+^ and reduced GSH induced by NMDA and GLU treatment increases ROS production. The accumulation of ROS eventually leads to ferroptosis in VECs, which can be effectively reversed by potent ferroptosis inhibitors, Fer-1 or Lip-1, or an iron chelation, DFO. In contrast, we confirmed the role of NMDAR using MK-801, a potent NMDAR inhibitor, in inhibiting the stimulation of ferroptosis, suggesting that NMDAR restriction may have a potential effect on ferroptosis in VECs induced by NMDA or GLU.

SLC7A11 is a component of the cystine/glutamate antiporter system [44] and inhibition of SLC7A11 suppresses the production of GSH, leading to the inactivation of GPX4. Therefore, SLC7A11 is a key factor in ferroptosis [45, 46]. We found that GPX4 is expressed at low levels after NMDAR activation in HUVECs. In contrast, SLC7A11 is highly expressed, resulting in GSH depletion and cellular ferroptosis-like death, suggesting that VECs may clear ROS by GPX4 when NMDAR is activated in the process of cell death. The upregulation of SLC7A11 after NMDA and GLU exposure may be interpreted as a compensatory mechanism for defense measures. It is speculated that additional feedback is activated to initiate protective measures that promote excessive glutamate release.

It has been reported that the PP2A/AMPK pathway is involved in ferroptosis. AMPK regulates metabolism, mitochondrial homeostasis, and autophagy [47]. It is activated when glucose is absent, turning on an energy stress-protective program against ferroptosis that involves impaired biosynthesis of polyunsaturated fatty acids, which are essential for lipid peroxidation-driven ferroptosis [48]. The present study identified a new role of AMPK in ferroptosis by activating NMDAR via the PP2A/AMPK/HMGB1 pathway. We demonstrated that NMDAR activation activated PP2A by dephosphorylation of the Tyr307 site on PP2Ac, thereby dephosphorylating AMPK and further increasing the expression of HMGB1 to induce ferroptosis in HUVECs. PP2A regulates protein synthesis by negatively regulating AMPK, which regulates HMGB1 and affects endothelial cell survival. HMGB1 is a DNA-binding non-histone protein implicated in DNA replication, transcription, and repair, and extracellular HMGB1 is considered a proinflammatory mediator as well as a ferroptosis regulator in human diseases [49]. Our findings confirmed that PP2A activity was promoted and phosphorylation was suppressed after NMDAR activation. Furthermore, phosphorylation of AMPK decreased, while the expression of HMGB1 increased. Inhibition of PP2A with LB-100, activation of AMPK, or knockdown of HMGB1 reversed these changes, indicating that PP2A/AMPK/HMGB1 play a crucial role in NMDAR activation-induced ferroptosis.

## Conclusion

While the specific mechanism is different, the AMPK signaling pathway partially mediates the process of ferroptosis. Our study proves that NMDA and GLU activate PP2A and inhibit AMPK, further promoting HMGB1 and mediating ferroptosis. In this study, the mechanisms of NMDAR activation-induced ferroptosis in endothelial cells were investigated, and some of the conclusions were confirmed in vivo. This study aimed to provide a new perspective on the treatment of endothelial cell death as well as a specific theoretical basis and idea for developing new drugs to protect against endothelial ferroptosis.

## Materials and methods

### Cell culture and exposure

Human umbilical vein endothelial cells (HUVECs) were obtained from ScienCell Research Laboratories (San Diego, CA, USA) and cultured in plates pre-coated with 0.2% gelatin in endothelial cell medium supplemented with 5% fetal bovine serum, 1% penicillin/streptomycin, and 1% endothelial cell growth supplement (ScienCell) at 37 °C with 5% CO_2_.

For each experiment using cultured HUVECs, cells were seeded in the incubator and cultured to 70–80% confluence, then exposed to L-glutamic acid (GLU, 20 mM, Cat#: G8415, Sigma-Aldrich, St. Louis, MO, USA) or N-Methyl-D-aspartic acid (NMDA, 1 mM, Cat#: HY-17551, MedChemExpress, Shanghai, China) for 24 h in the absence or presence of Ferrostatin-1 (Fer-1, 10 μM, Cat#: HY-100579, MedChemExpress), Liproxstatin-1 (Lip-1, 2 μM, Cat#: HY-12726, MedChemExpress), LB-100 (5 μM, Cat#: S7537, Sellechchem, Houston, TX, USA), Acadesine (AUCAR, 2 μM, Cat#: HY-13417, MedChemExpress), Deferoxamine (DFO, 10 μM, Cat#: HY-B0988, MedChemExpress), and Erastin (5 μM, Cat#: HY-15763, MedChemExpress) for 24 h. These plating conditions were used in all experiments, unless mentioned otherwise.

### Cell viability

Cell viability was assessed using the 3-(4,5-dimethyl-2-thiazolyl)-2,5-diphenyl-2H-tetrazolium bromide (MTT) assay (Solarbio Technology, Beijing, China), as described previously. Briefly, HUVECs were seeded in 96-well plates and cultured for 24 h. Cells were pretreated with the corresponding inhibitors or small interfering RNA (siRNA), and then exposed to a designated concentration of NMDA and GLU for the indicated times. After treatment, the cells were incubated with 0.5 mg/mL MTT for 4 h and resuspended in 150 μL DMSO (Sigma-Aldrich, CA, USA). The absorbance was measured at 495 nm using an Infinite M200 Pro NanoQuant (TECAN, Switzerland).

### Immunofluorescence for Fe^2+^ analysis

FerroOrange (Dojindo Laboratories, Kumamoto, Japan) was used to determine Fe^2+^ levels by immunofluorescence staining. Briefly, HUVECs were plated on confocal culture dishes and treated with GLU or NMDA in the absence or presence of Fer-1, Lip-1, and DFO for 24 h. Cells were washed three times with phosphate-buffered saline (PBS), and images were captured using a scanning confocal microscope (EVOS™ FL Auto 2 Imaging System, Thermo Fisher Scientific, MA, USA).

### Glutathione (GSH) measurement

The treated HUVECs were harvested and lysed by sonication at 0 °C for 20 min, followed by centrifugation at 15,000 × *g* at 4 °C for 10 min. The cleared supernatant was prepared to detect the total protein concentration using a Protein Assay Kit (Thermo Fisher Scientific) and to measure the amount of GSH using the GSSG/GSH Quantification Kit. To remove proteins from samples, 1/6 volume of 5% 5-sulfosalicylic acid dihydrate (Wako Pure Chemical Corporation, Japan) in distilled water was used. Samples were centrifuged at 15,000 × *g* at 4 °C for 10 min, and the supernatant was used for the GSH assay. The protein and GSH levels in the samples were detected according to the manufacturer’s instructions by measuring the absorbance at 405 nm using a plate reader. Values for total GSH levels were calculated, corrected for protein concentration in the same sample, and normalized to the control.

### Measurement of malondialdehyde (MDA) and lipid peroxidation (LPO) levels

MDA (A003-1; Nanjing Jiancheng Bioengineering Institute, Nanjing, China) and LPO (A106-1, Nanjing Jiancheng Bioengineering Institute) in vascular tissues or cells were measured using commercial kits, according to the manufacturer’s instructions. Briefly, cells (1 × 10^6^) were collected in 200 μL of lysis buffer and homogenized on ice. Subsequently, an MDA test solution (1000 μL) was added to each experimental sample or vial containing the standard sample to form an MDA-TBA adduct, followed by incubation for 40 min at 95 °C. For LPO analysis, 800 μL of the LPO test solution was added to each experimental sample or vial containing the standard sample and then incubated for 60 min at 45 °C. The mixture was cooled to 25 °C in an ice bath and centrifuged at 4000 rpm for 10 min. The insoluble material was removed, and 250 μL of each reaction mixture was pipetted into 96-well plates for colorimetric assays to measure the absorbance of MDA and LPO at 530 nm and 586 nm, respectively, with an Infinite M200 Pro NanoQuant (TECAN).

### siRNA

siRNA was used to silence the expression of specific genes in HUVECs. The cells at 60–70% confluence were transfected with specific siRNA duplexes (60 nM, Santa Cruz Biotechnology, CA, USA) using Lipofectamine RNAiMAX Reagent (Thermo Fisher Scientific) following the manufacturer’s instructions. After 48 h of transfection with control siRNA, HMGB1 siRNA, PP2A siRNA, and AMPK siRNA (Santa Cruz Biotechnology), the cells were incubated with GLU or NMDA for the indicated times and then collected for western blot analysis and cell viability.

### Animal experiments

The animal study protocol was approved by the Animal Care and Ethics Committee of Xiamen University. Male C57BL/6 mice, aged 6–8 weeks, were obtained from the Beijing Vital River Laboratory Animal Technology Co., Ltd. (Beijing, China) and raised in the Laboratory Animal Center of Xiamen University. A total of 64 mice were randomly assigned to eleven groups (Grouping according to the complete random number method; experimental operation and data analysis were according to animal No. and not grouped information.): (1) control (*n* = 8); (2) GLU (1 g/kg, n = 8); (3) GLU + LB-100 (*n* = 8, 2 mg/kg); (4) GLU + AICAR (*n* = 8, 100 mg/kg); (5) GLU + MK-801 (0.5 mg/kg, n = 8); (6) GLU + Glycyrrhizin acid (GLY, n = 8, 20 mg/kg, Cat#: HY-N0184, MedChem Express); (7) NMDA (75 mg/kg, n = 8); (8) NMDA + LB-100 (2 mg/kg, n = 8); (9) NMDA + AICAR (100 mg/kg, n = 8); (10) NMDA + MK-801(0.5 mg/kg, n = 8); and (11) NMDA + GLY (20 mg/kg, n = 8). Animals were intraperitoneally injected with different compounds twice daily for ten consecutive days, and control animals received equal volume of vehicle (0.9% saline) or DMSO subcutaneously. All animals were maintained at room temperature (23 ± 2 °C) with a 12 h light/dark cycle and free access to a basic diet and water. When the animals were sacrificed at the end of the experiment, vascular tissues were collected for further data analysis.

### Detection of intracellular ferrous ions and lipid reactive oxygen species (LOS)

Cell fluorescence levels of total intracellular ferrous ion and LOS were detected using FerroOrange (10 μM) and Liperfluo (1 μM), respectively. When cells were treated with the test compounds at the indicated times, cells in 6-well dishes were resuspended in 500 ml of fresh Hank’s balanced salt solution (HBSS) for Liperfluo evaluation for 30 min at 37 °C in a culture incubator, and cells in 24-well plates were used for FerroOrange staining in HBSS for 30 min at 37 °C. After washing three times, intracellular FerroOrange fluorescence and LOS were determined in the single-cell suspension using a flow cytometer (Beckman Coulter, USA) with an excitation wavelength of 488 nm and an emission wavelength of 525 nm. Increased percentages of LOS levels were determined by quantifying and calculating the ratio of 488 nm channel intensity in treated groups compared to that in control groups.

### Iron content assay

Vascular tissues isolated from mice or cultured HUVECs were homogenized in saline or PBS. The protein concentration was measured using the BCA Protein Assay Kit. The levels of iron in the blank (ddH_2_O), iron standard solution, and test samples (supernatant) were examined using an Iron Assay Kit (TC1015; Leagene Biotechnology, Beijing, China) according to the manufacturer’s instructions. The reaction mixture was then incubated at room temperature for 15 min. The absorbance was measured at 562 nm using a microplate reader (Infinite M200 Pro Nano Quant, TECAN).

### PP2A activity assay

Vascular tissues isolated from mouse or cultured HUVECs were homogenized in saline or PBS, followed by centrifugation. PP2A activity was measured according to the manufacturer’s protocol (R&D Systems, DYC3309-2).

### Western blot analysis

Western blot analysis was used to determine the expression of specific proteins in the vascular tissues and cultured HUVECs. Proteins of tissue homogenate lysates or HUVECs lysates prepared in sodium dodecyl sulfate (SDS) lysis buffer were extracted with RIPA buffer supplemented with protease and phosphatase inhibitors on ice, and protein concentration was determined using the BCA Protein Assay Kit (Solarbio), as described previously. SDS-PAGE and polyvinylidene difluoride membranes (Bio-Rad, Hercules, CA, USA) were used to separate proteins. The membranes were blocked with 5% fat-free dry milk in 0.1% Tris-buffered saline with Tween for 2 h and then probed with different primary antibodies at 4 °C overnight, including anti-PTGS2 (abcom, ab283574, 1:1000), Anti-Glutathione Peroxidase 4 antibody (abcom, ab125066, 1:1000), Anti-Transferrin Receptor antibody (abcom, ab214039, 1:1000), Anti-xCT antibody (abcom, ab175186, 1:1000), Anti-PP2A antibody (abcom, ab32104, 1:1000), Anti-Phospho-PP2A antibody (santacruz, sc-271903, 1:1000), Anti-AMPKα antibody (CST, #2532, 1:1000), Anti-Phospho-AMPKα antibody (CST, #2535, 1:1000), Anti-HMGB1 antibody (abcom, ab79823, 1:10000) and Anti-β-Actin antibody (santacruz, sc-47778, 1:1000). After washing, the membranes were incubated with the secondary antibody (1:10000) for 1 h at room temperature. Blots were visualized using ECL ™ reagents (Advansta, Menlo Park, CA, USA). Protein signals were captured using the FluorChem E chemiluminescence detection system (ProteinSimple, San Jose, CA, USA). All western blots were performed at least five times. The signal intensity of the immunoreactive bands was quantified using ImageJ software (NIH, Bethesda, MD, USA) and normalized to that of β-actin in each sample.

### AMPK activity assay

VECs cultured with NMDA and GLU for 24 h were collected and prefixed in 2.5% glutaraldehyde phosphate (0.1 M, pH 7.4) overnight at 4 °C, post-fixed in 2% buffered osmium tetraoxide, and then embedded in Epon812 (Merck, NJ, USA), followed by dehydration. Ultrathin sections (60 nm thick) were cut and stained with uranyl acetate as well as lead citrate. Images were examined using a Hitachi HT7800 TEM instrument (Tokyo, Japan).

### Statistical analysis

Statistical analyses were performed using GraphPad Prism 9.0 (GraphPad Software Inc., San Diego, CA, USA). All results are presented as the mean ± SEM from at least three separate experiments unless otherwise described. One-way analysis of variance followed by the Bonferroni post-hoc test was used to compare the groups. The variance was similar among the groups that were being statistically compared. Statistical significance was set at *P* < 0.05. The exact sample size was provided in the figure legend.

### Supplementary information

NMDARs Activation Regulates Endothelial Ferroptosis via the PP2A-AMPK-HMGB1 axis

## Data Availability

All the data and experimental details in this article may be obtained from the corresponding author upon reasonable request.
