# Supplementary material for: NMDARs activation regulates endothelial ferroptosis via the PP2A-AMPK-HMGB1 axis
Source: Cell Death Discov. 2024 Jan 17;10:34. doi: 10.1038/s41420-023-01794-3 (PMC10794209; doi:10.1038/s41420-023-01794-3)
Supplement: Supplementary file 1 — NMDARs Activation Regulates Endothelial Ferroptosis via the PP2A-AMPK-HMGB1 axis [file 41420_2023_1794_MOESM1_ESM.docx]

Figure 1M, 1N

Lane 1: Control; Lane 2: GLU; Lane 3: GLU+Fer-1; Lane 4: GLU+Lip-1; Lane 5: GLU+DFO; Lane 6: none; Lane 7: Control; Lane 8: NMDA; Lane 9: NMDA+Fer-1; Lane 10: NMDA+Lip-1; Lane 11: NMDA+DFO


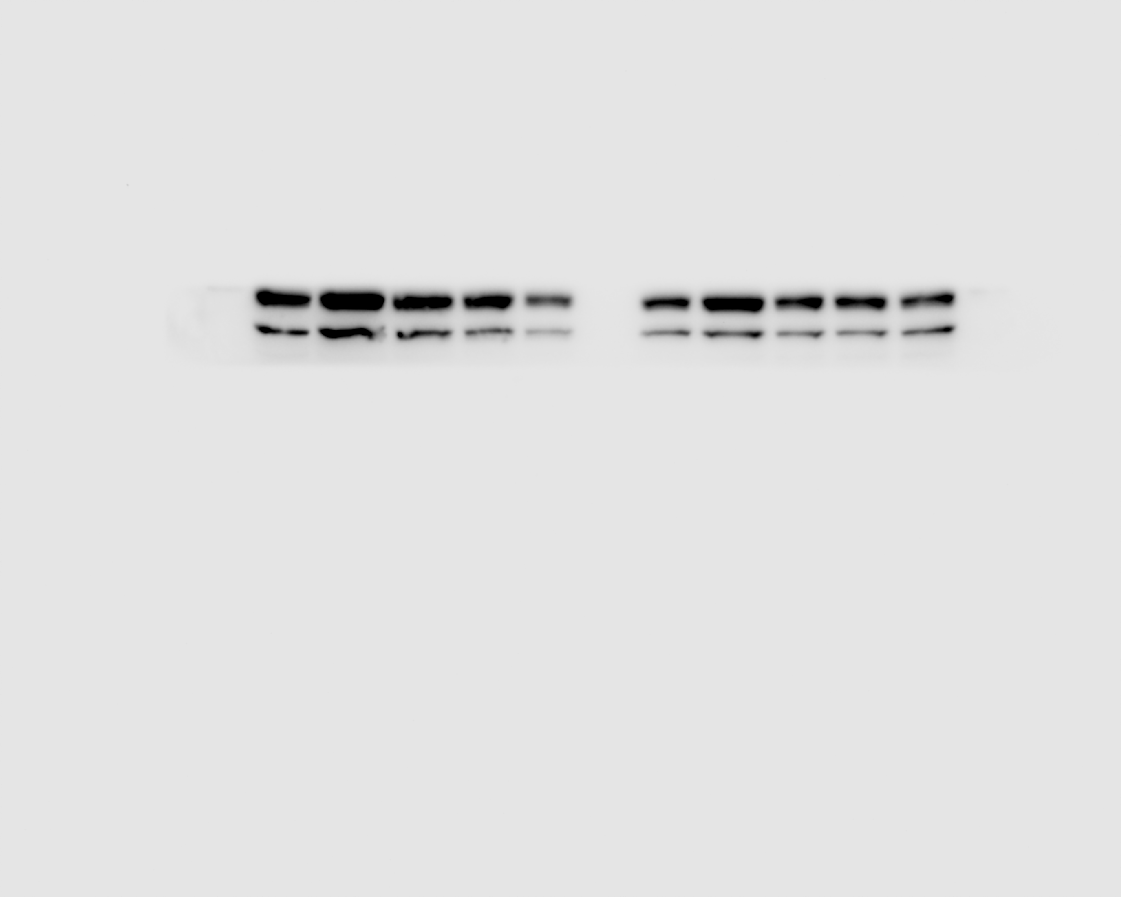


PTGS2 69KD


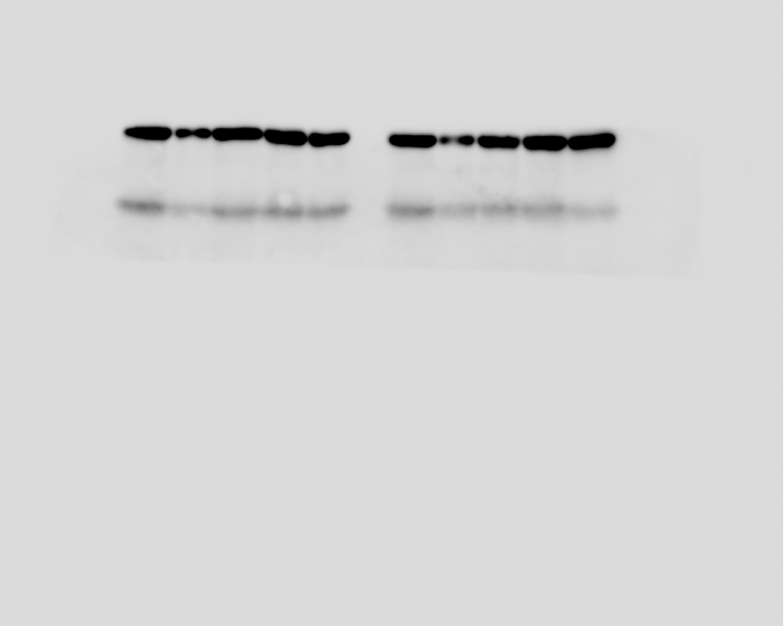


GPX4 20KD


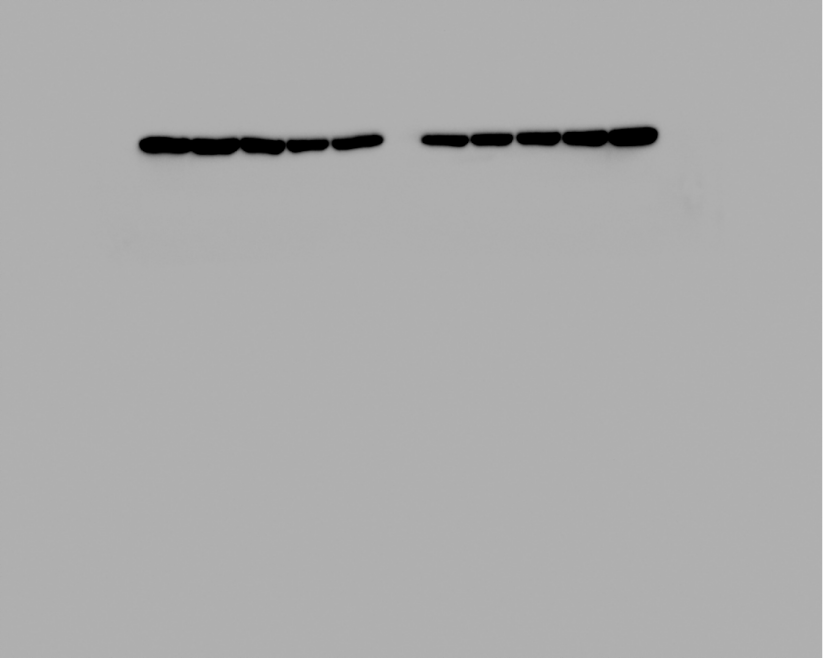


β-actin 43KD

20KD

Figure 2B

Lane 1: Control; Lane 2: GLU; Lane 3: NMDA; Lane 4: none; Lane 5: Control

Lane 6: GLU; Lane 7: NMDA; Lane 8: none; Lane 9: -; Lane 10: -; Lane 11: -Lane 12: -


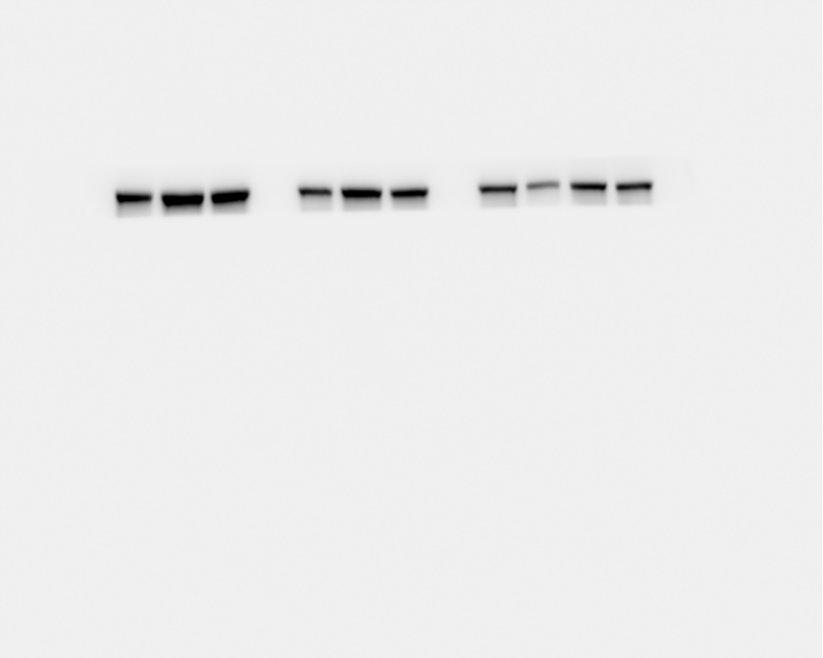


TfR1 90KD

20KD


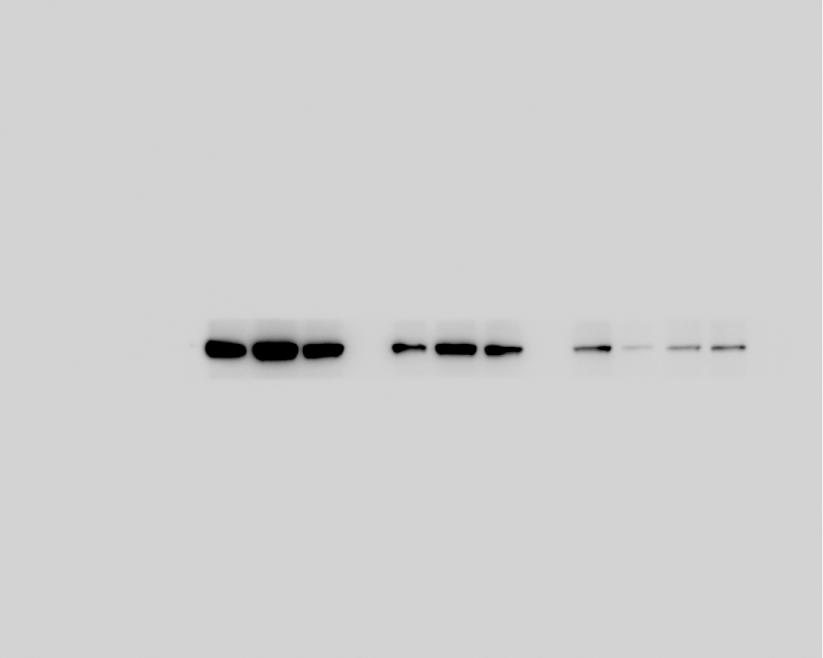


SLC7A11 55KD

20KD


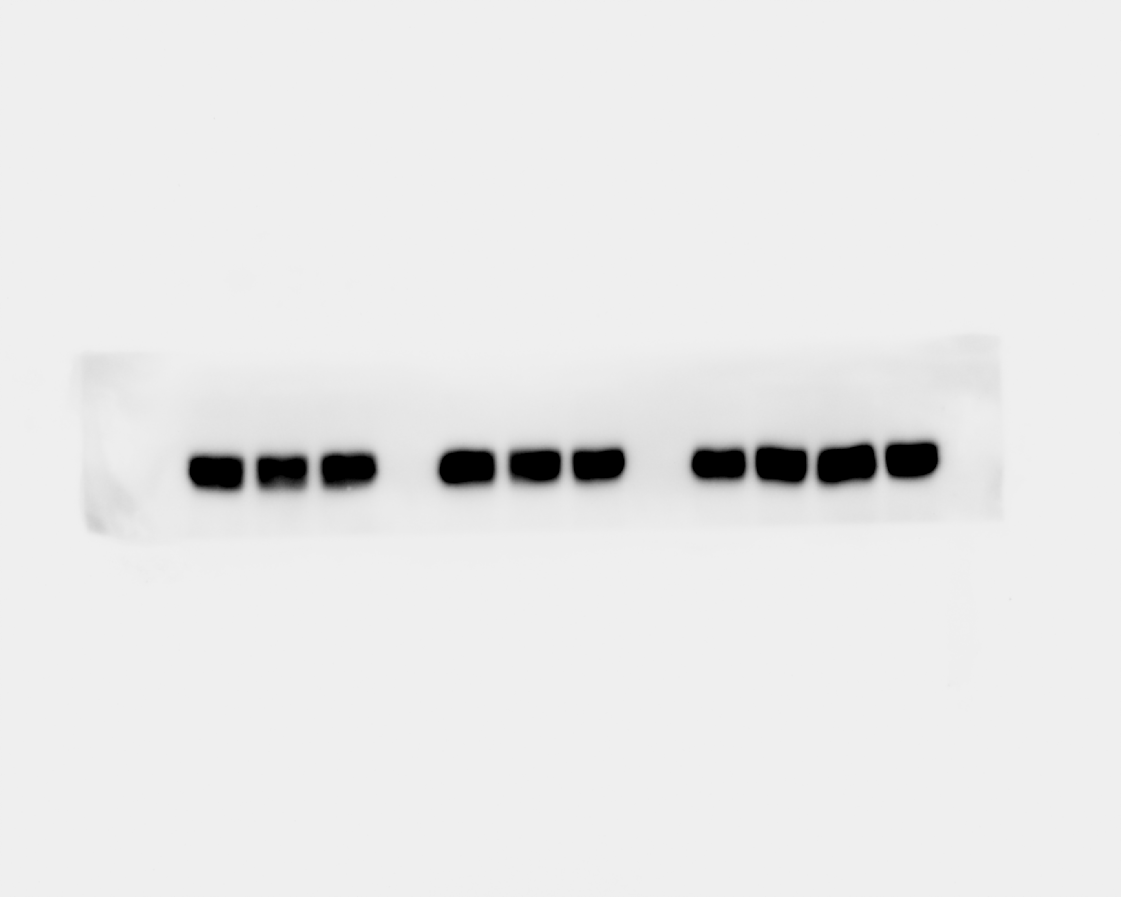


β-actin 43KD

20KD

Figure 2D

Lane 1: Control; Lane 2: GLU; Lane 3: NMDA; Lane 4: none; Lane 5: Control; Lane 6: GLU; Lane 7: NMDA; Lane 8: none; Lane 9: Control; Lane 10: GLU; Lane 11: NMDA


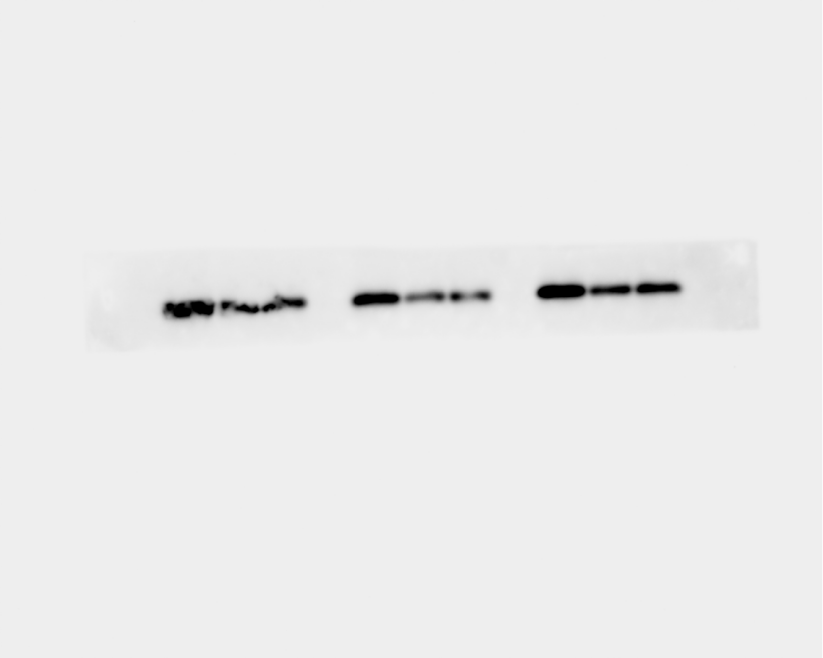


p-PP2A 36KD


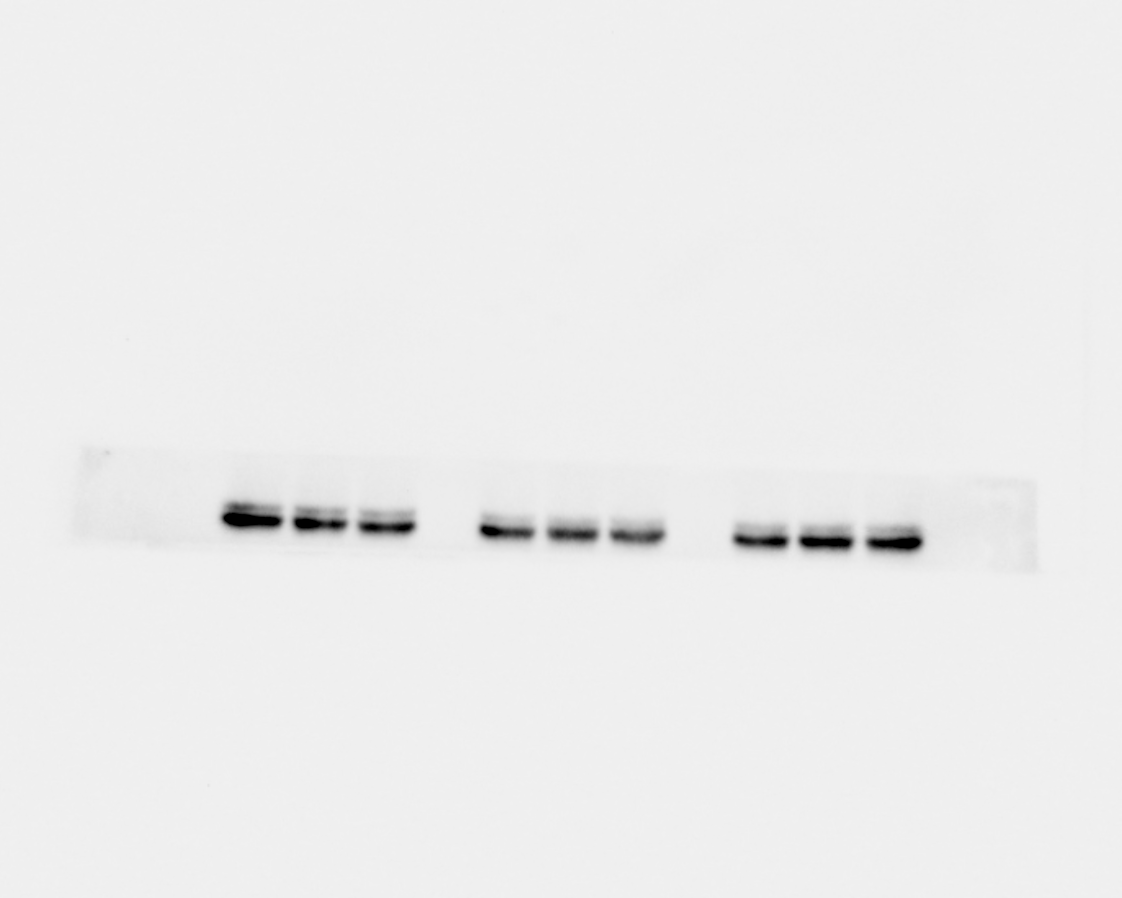


t-PP2A 36KD


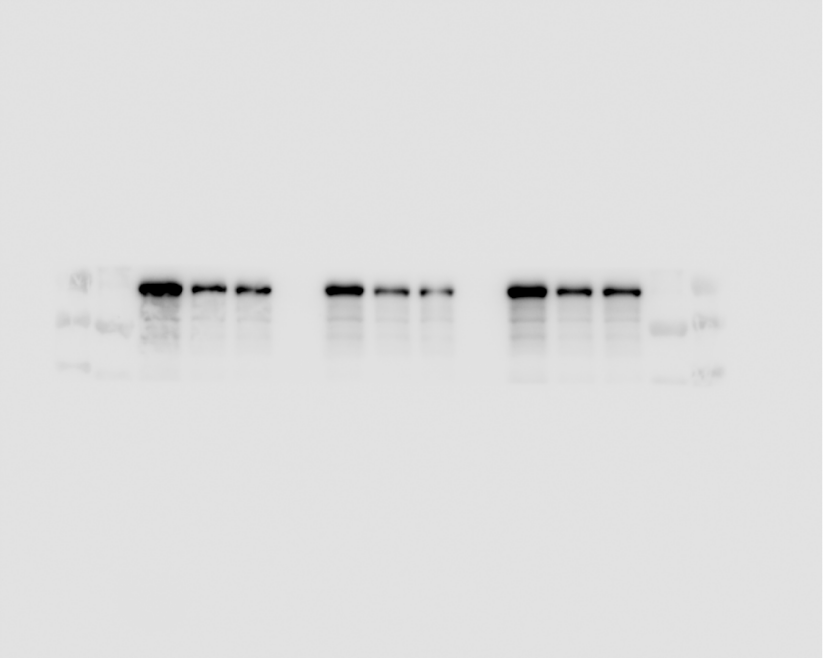


p-AMPK 62KD


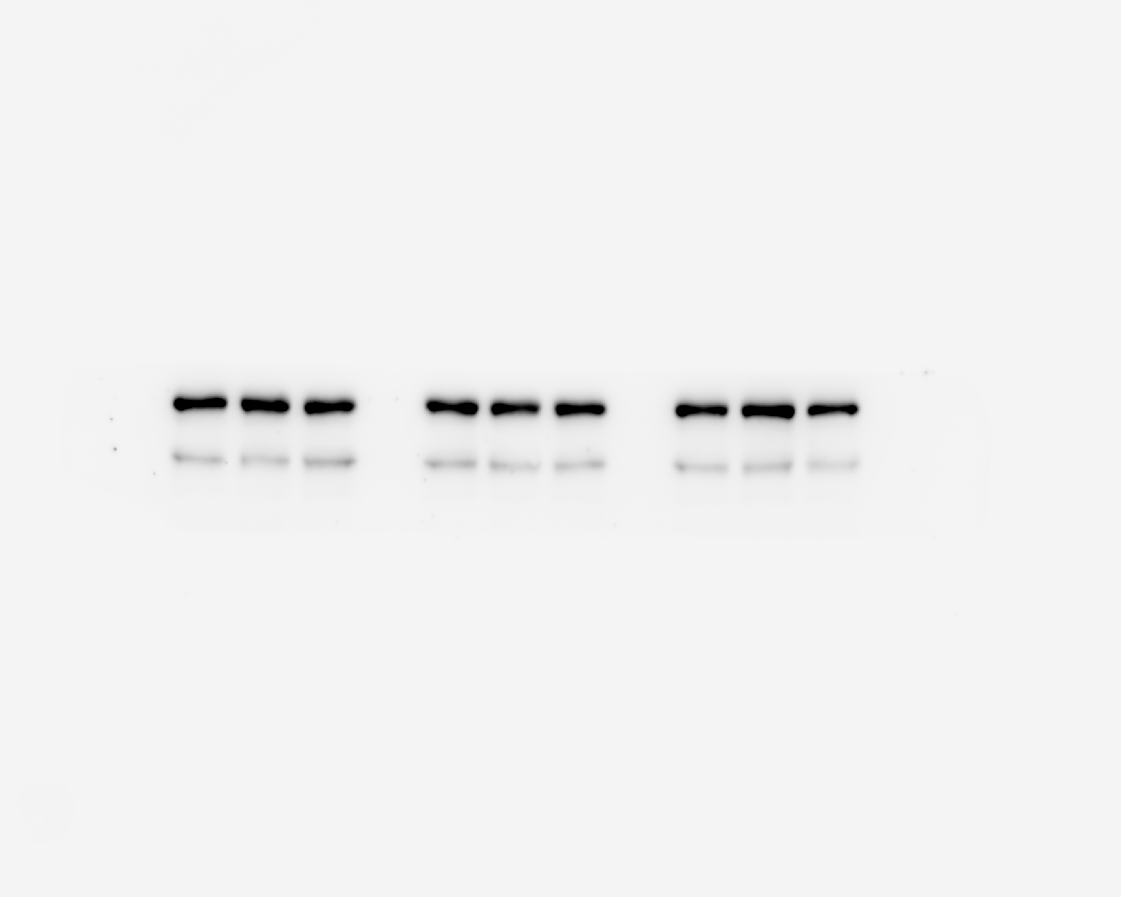


t-AMPK 62KD


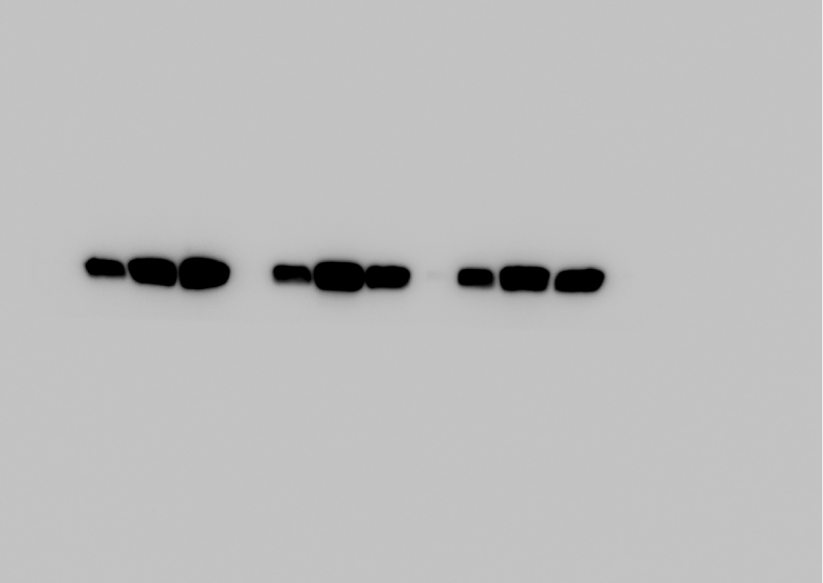


HMGB1 25KD


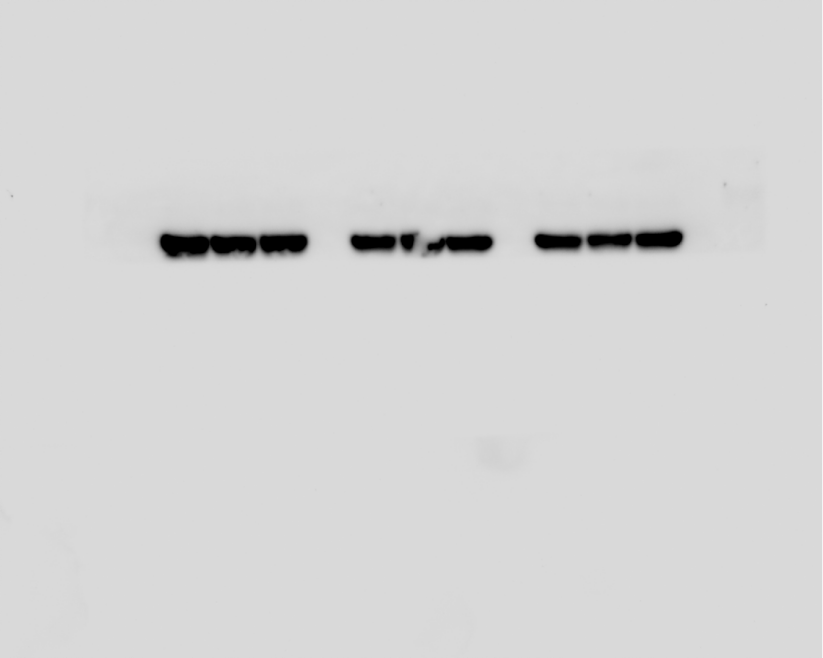


β-actin 43KD

20KD

Figure 2G

Lane 1: Control; Lane 2: GLU; Lane 3: GLU+LB-100; Lane 4: GLU+AICAR; Lane 5: none; Lane 6: Control; Lane 7: NMDA; Lane 8: NMDA+LB-100; Lane 9: NMDA+AICAR


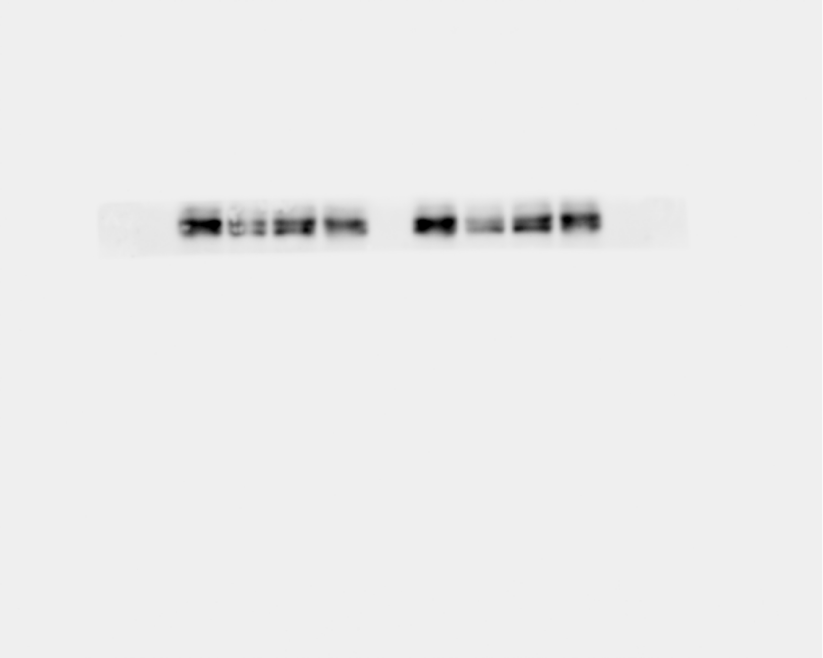


p-PP2A 36KD


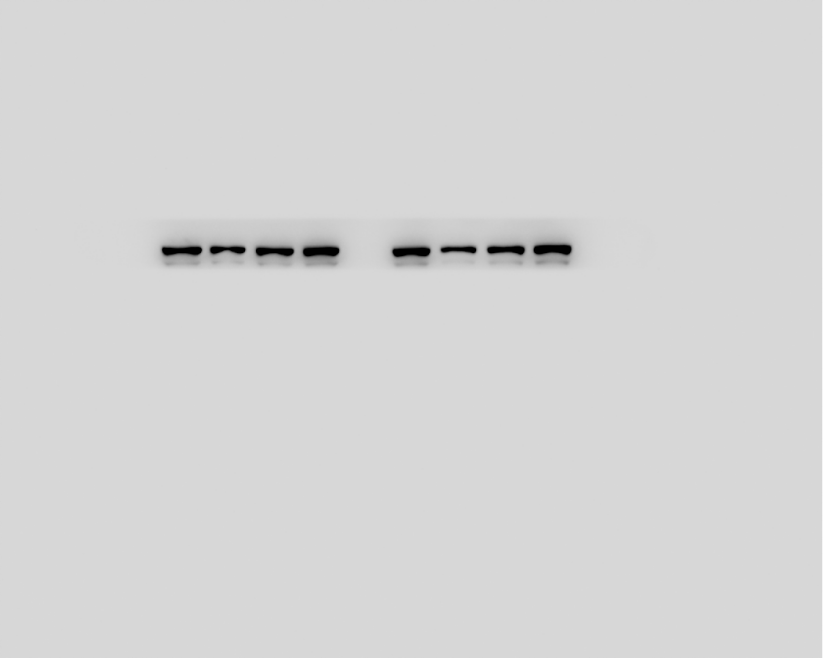


t-PP2A 36KD


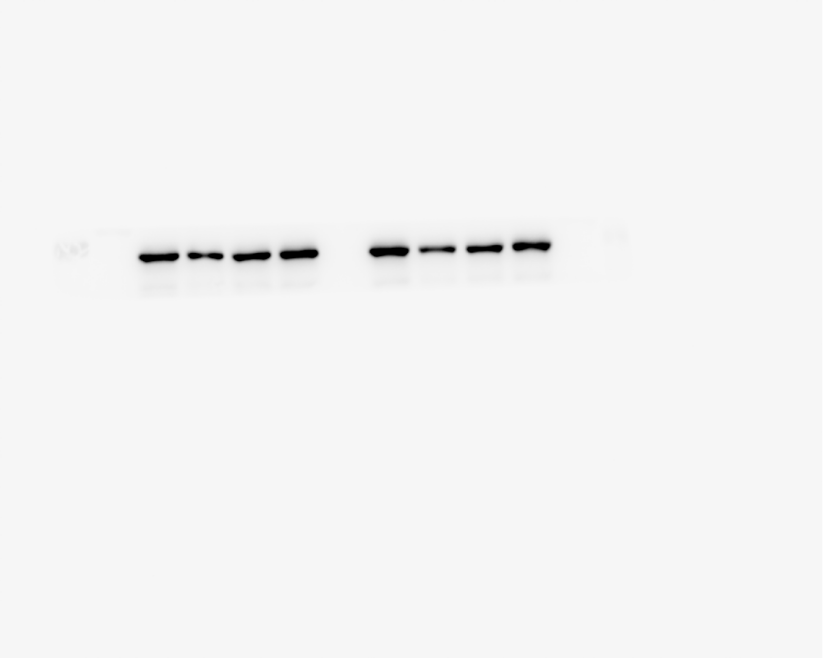


p-AMPK 62KD


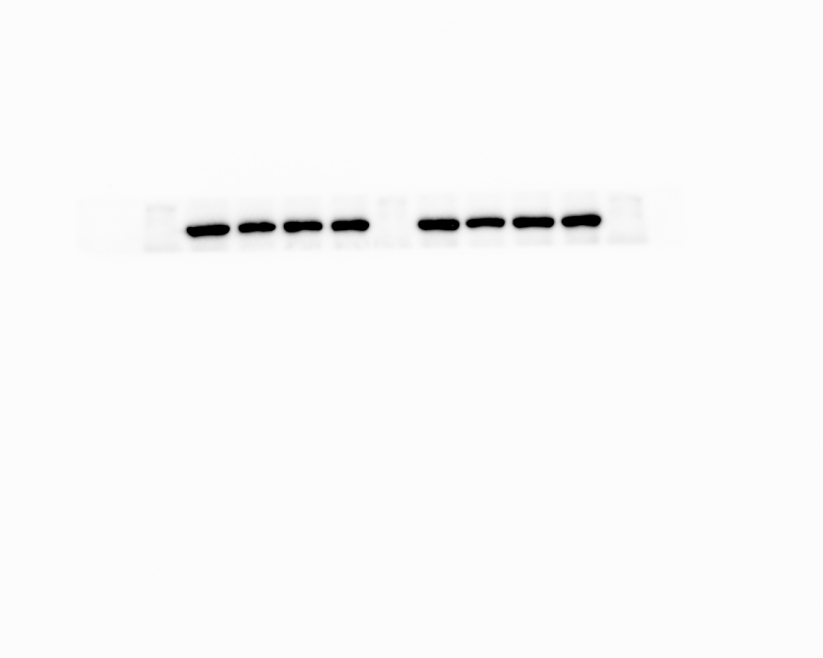


t-AMPK 62KD


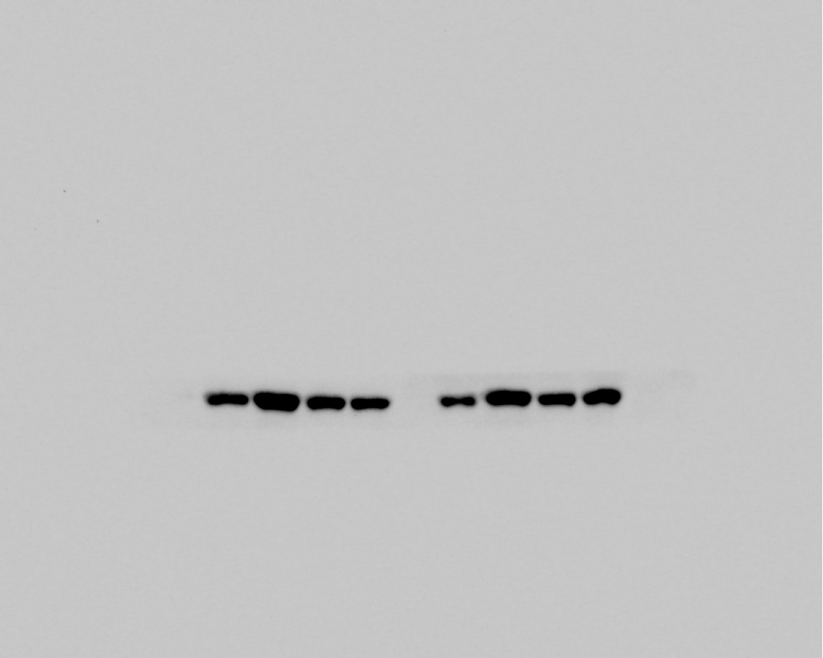


HMGB1 25KD


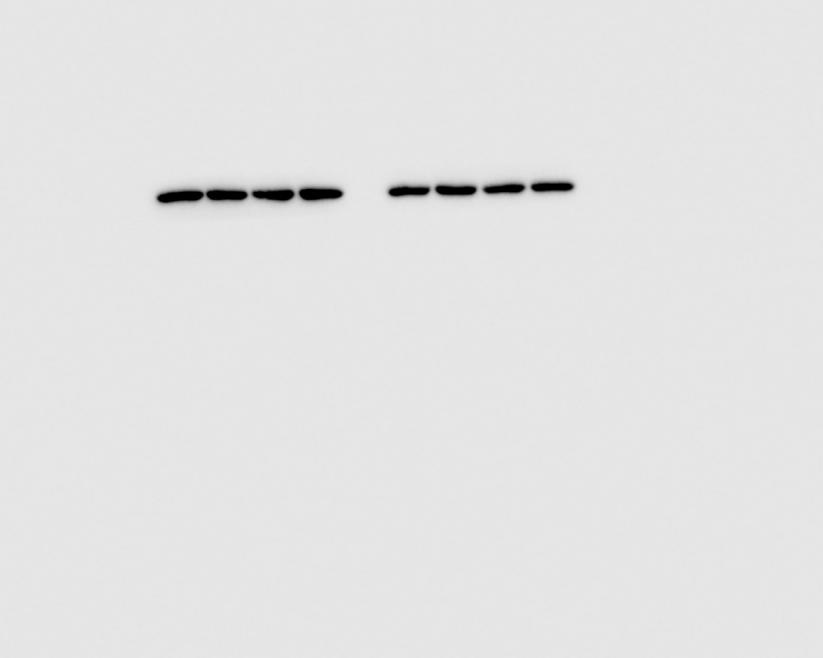


β-actin 43KD

20KD

Figure 3C

Lane 1: Vehicle + si-control; Lane 2: Vehicle + si-HMGB1; Lane 3: GLU + si-control; Lane 4: GLU + si-HMGB1; Lane 5: NMDA + si-control; Lane 6: NMDA + si-HMGB1; Lane 7: Vehicle + si-control; Lane 8: Vehicle + si-HMGB1; Lane 9: GLU + si-control; Lane 10: GLU+si-HMGB1; Lane 11: NMDA + si-control; Lane 12: NMDA+si-HMGB1


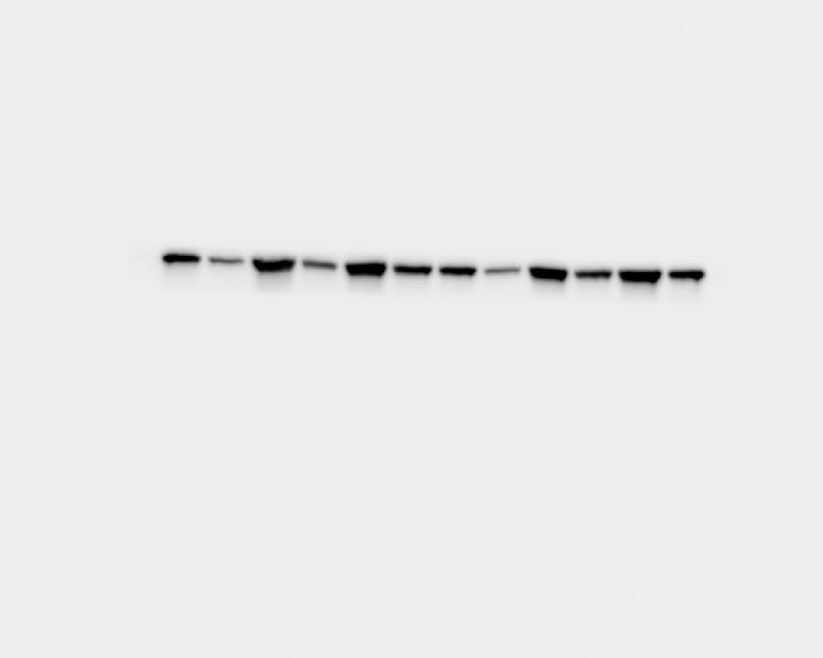


HMGB1 25KD


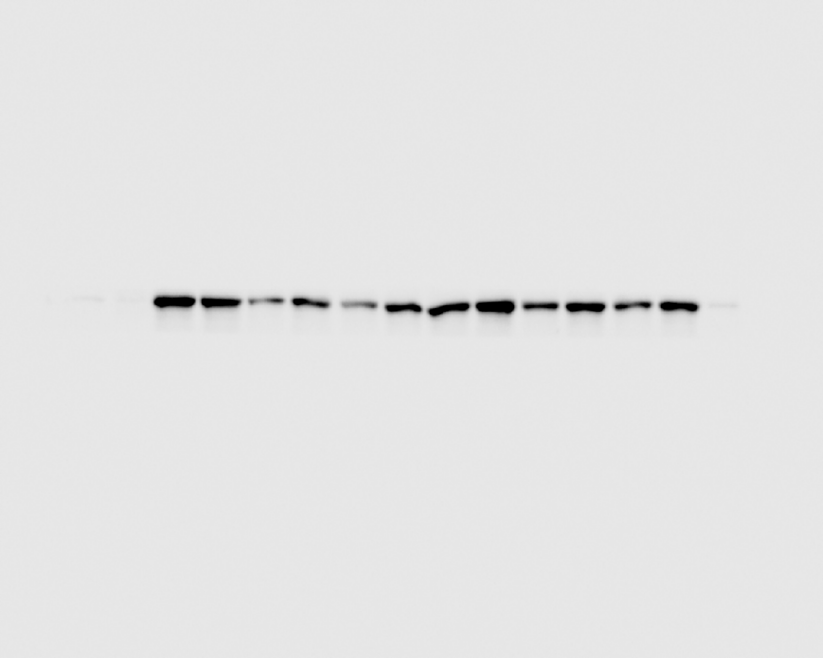


GPX4 20KD


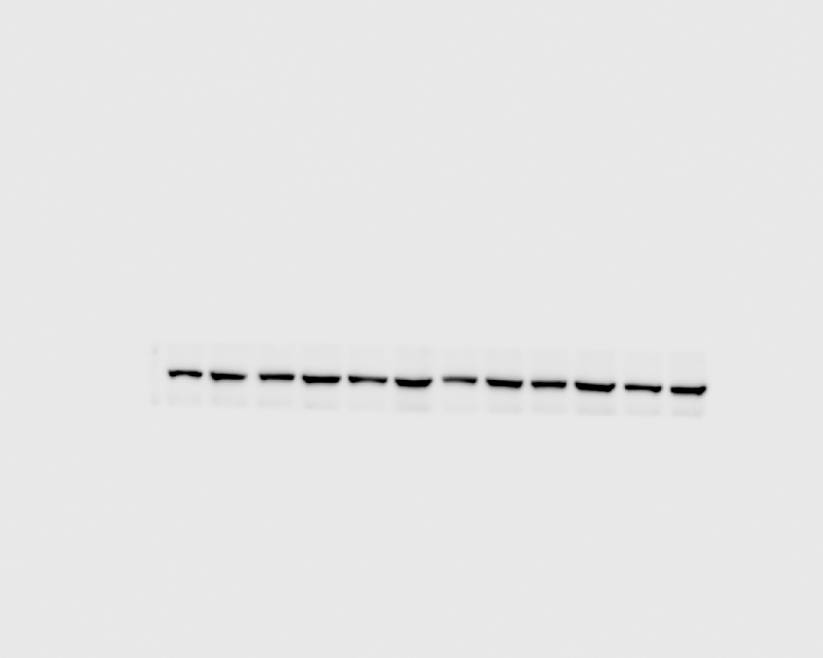


SLC7A11 55KD

20KD


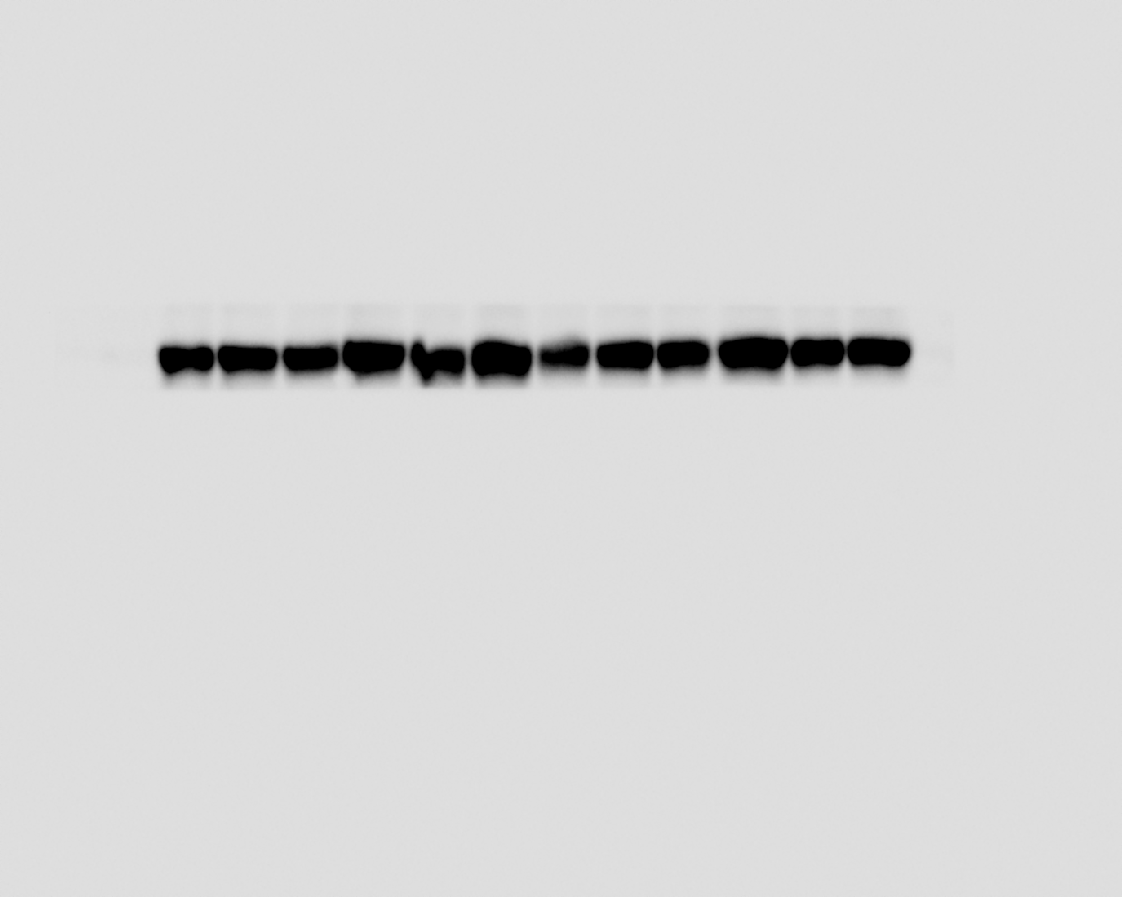


β-actin 43KD

20KD

Figure 4G

Lane 1: Control; Lane 2: Erastin; Lane 3: Erastin+MK801; Lane 4: Erastin+MK801; Lane 5: Erastin+MK801; Lane 6: none; Lane 7: Control; Lane 8: Rsl3; Lane 9: Rsl3+MK801

Lane 10: Rsl3+MK801; Lane 11: Rsl3+MK801


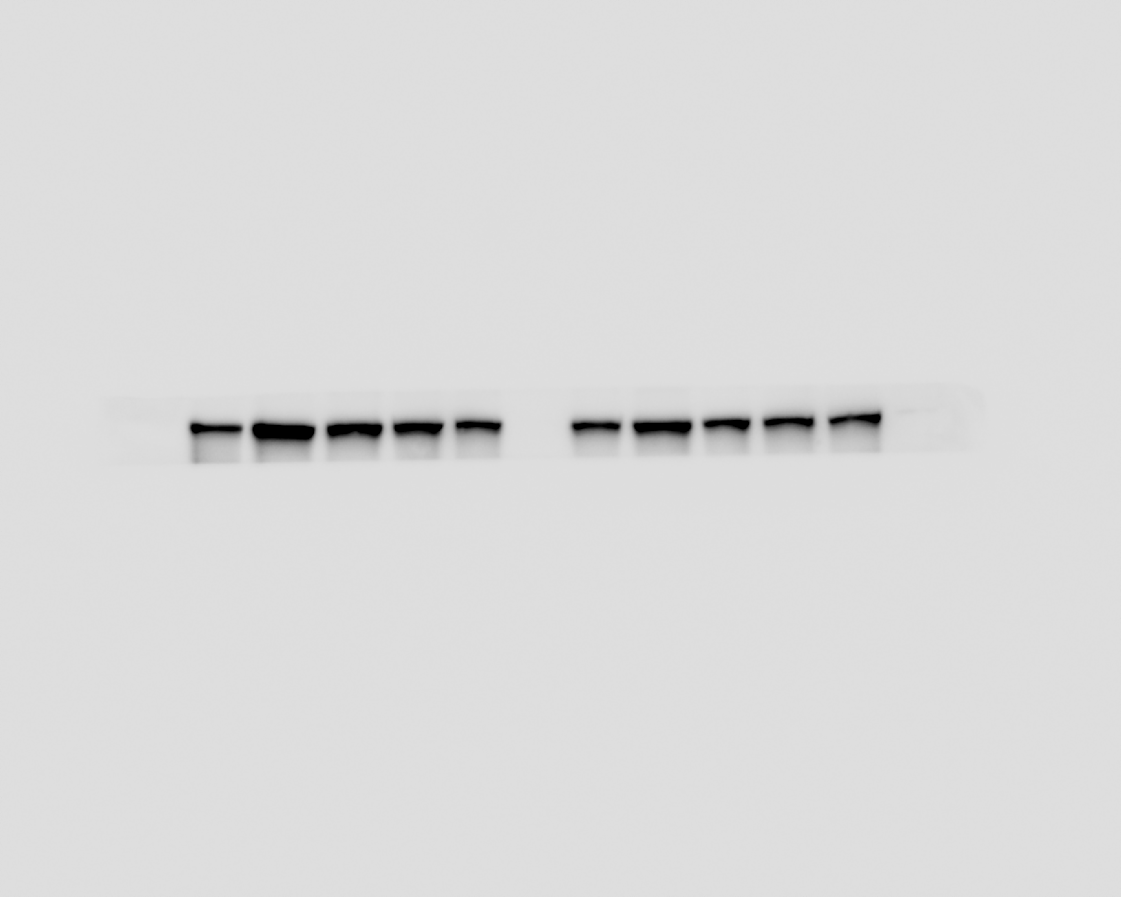


SLC7A11 55KD

20KD


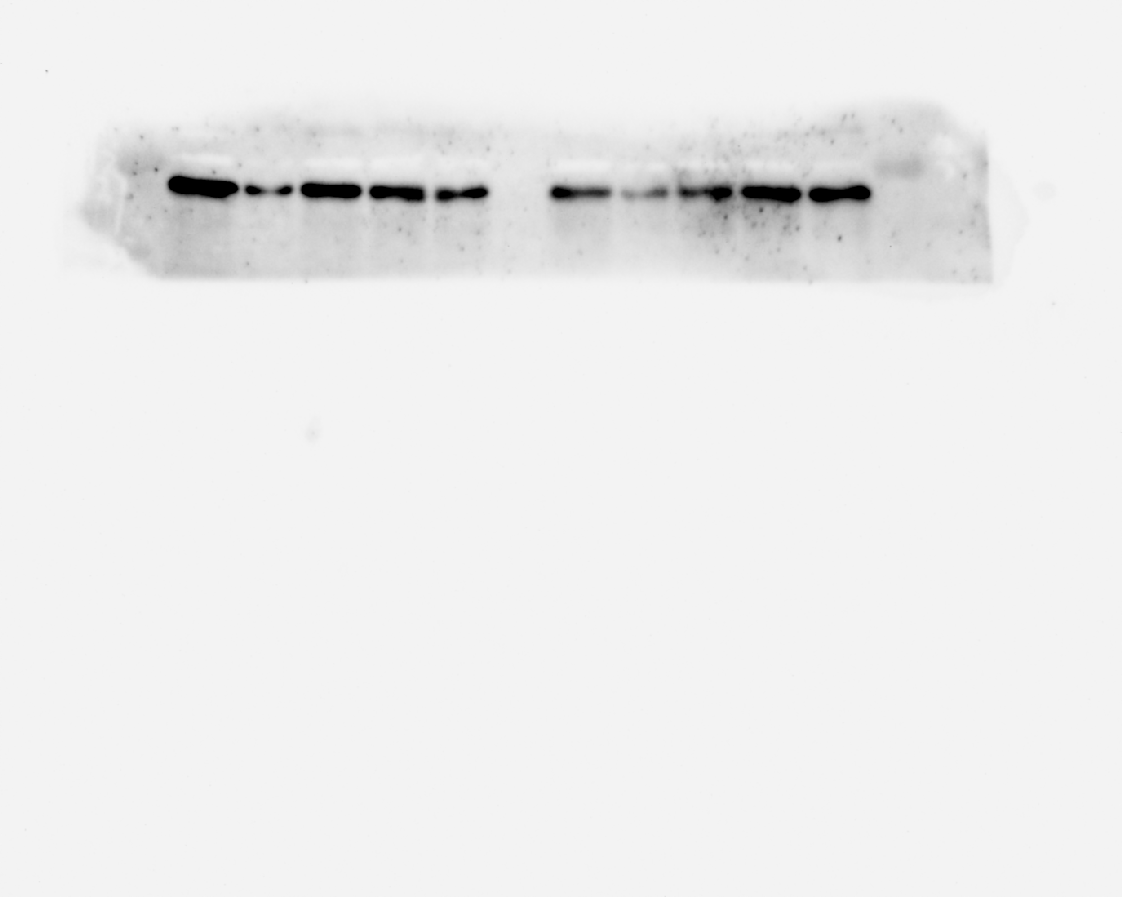


GPX4 20KD


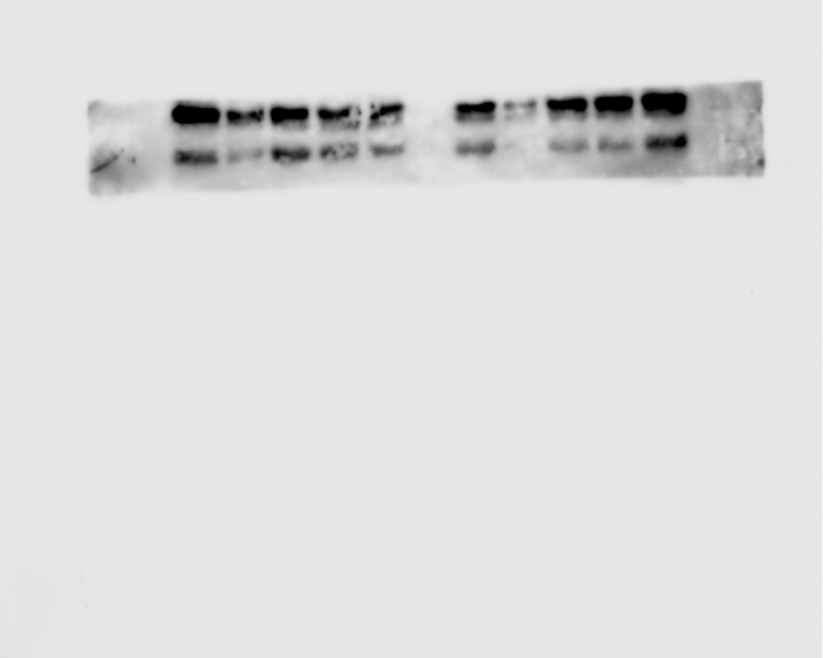


p-PP2A 36KD


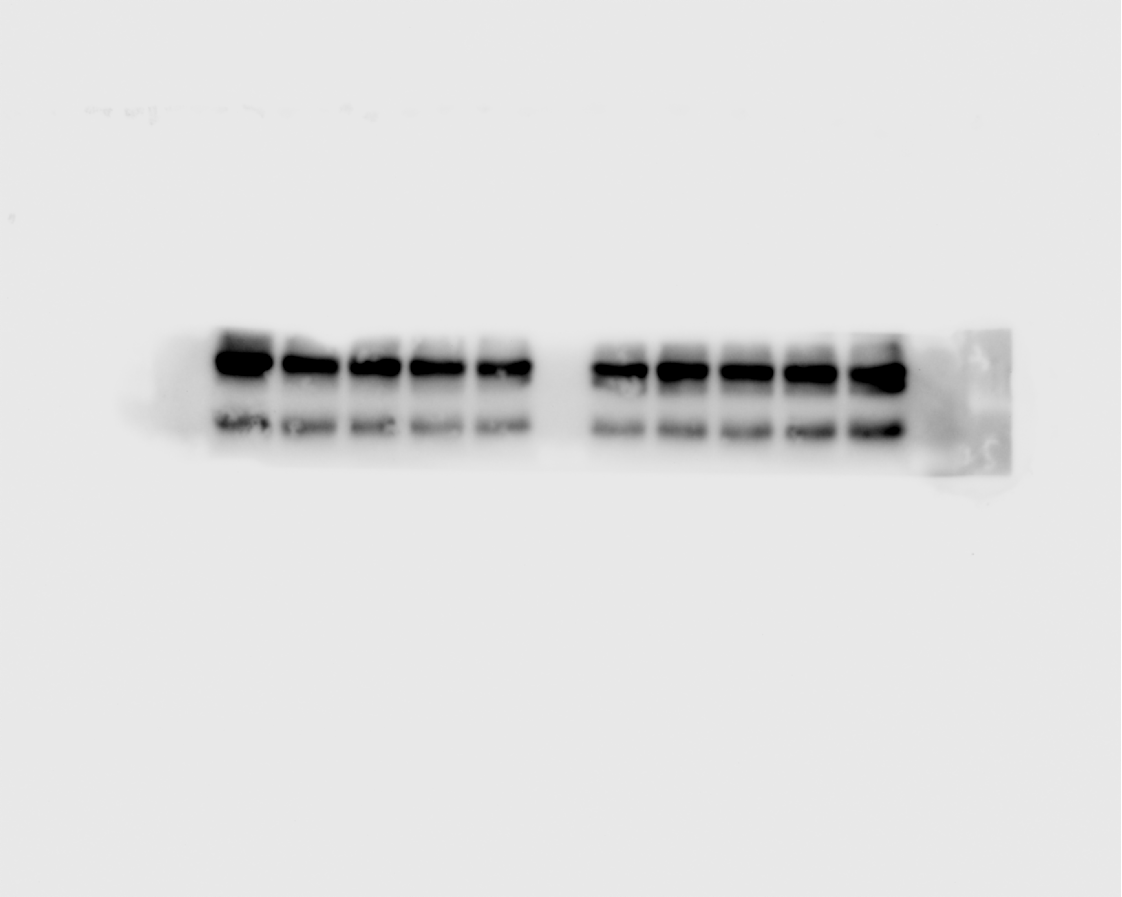


t-PP2A 36KD


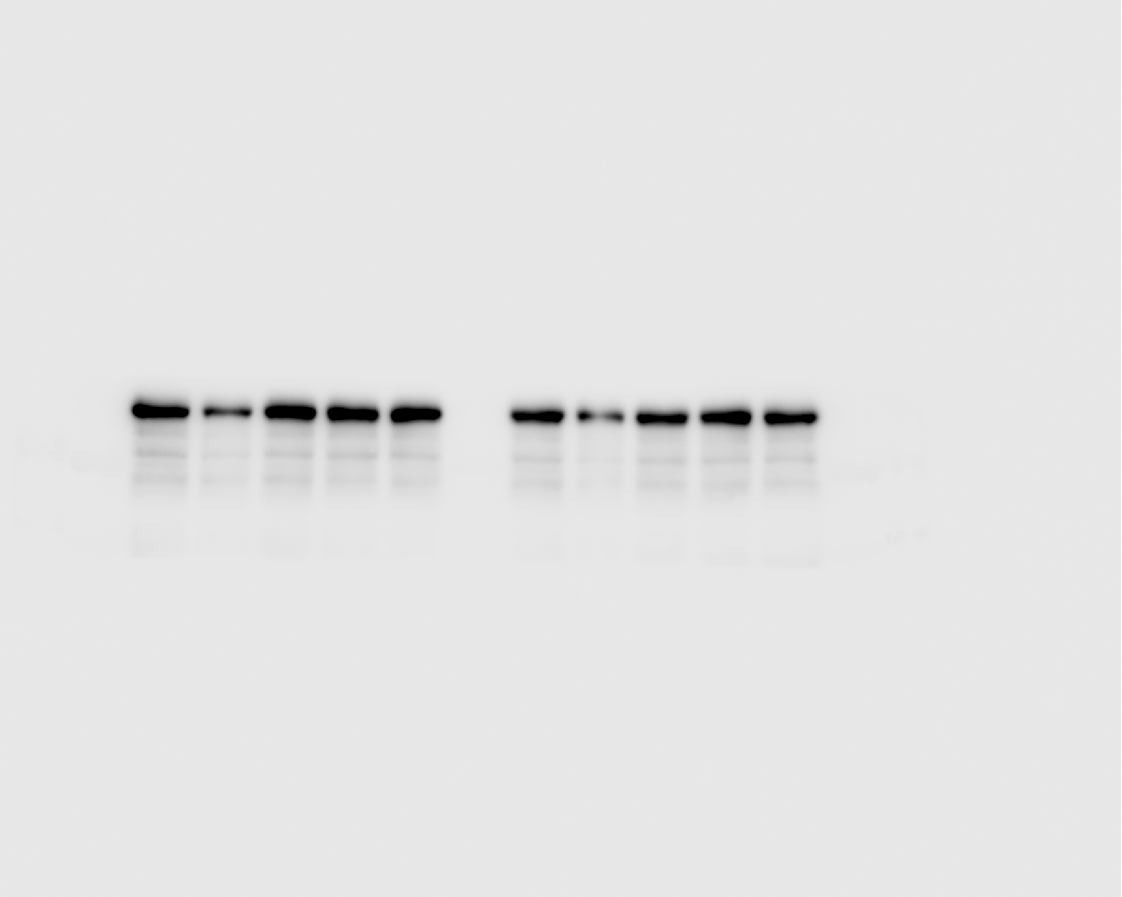


p-AMPK 62KD


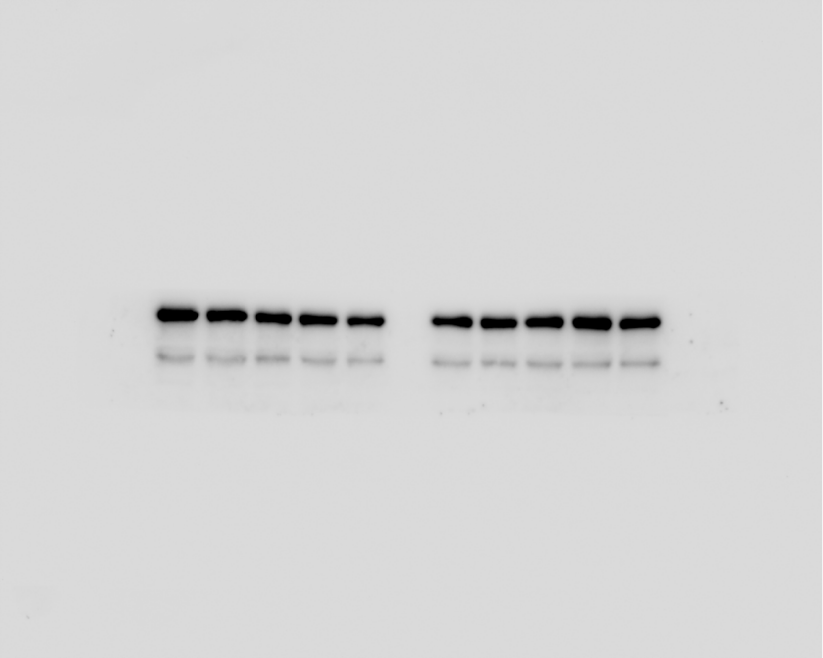


t-AMPK 62KD


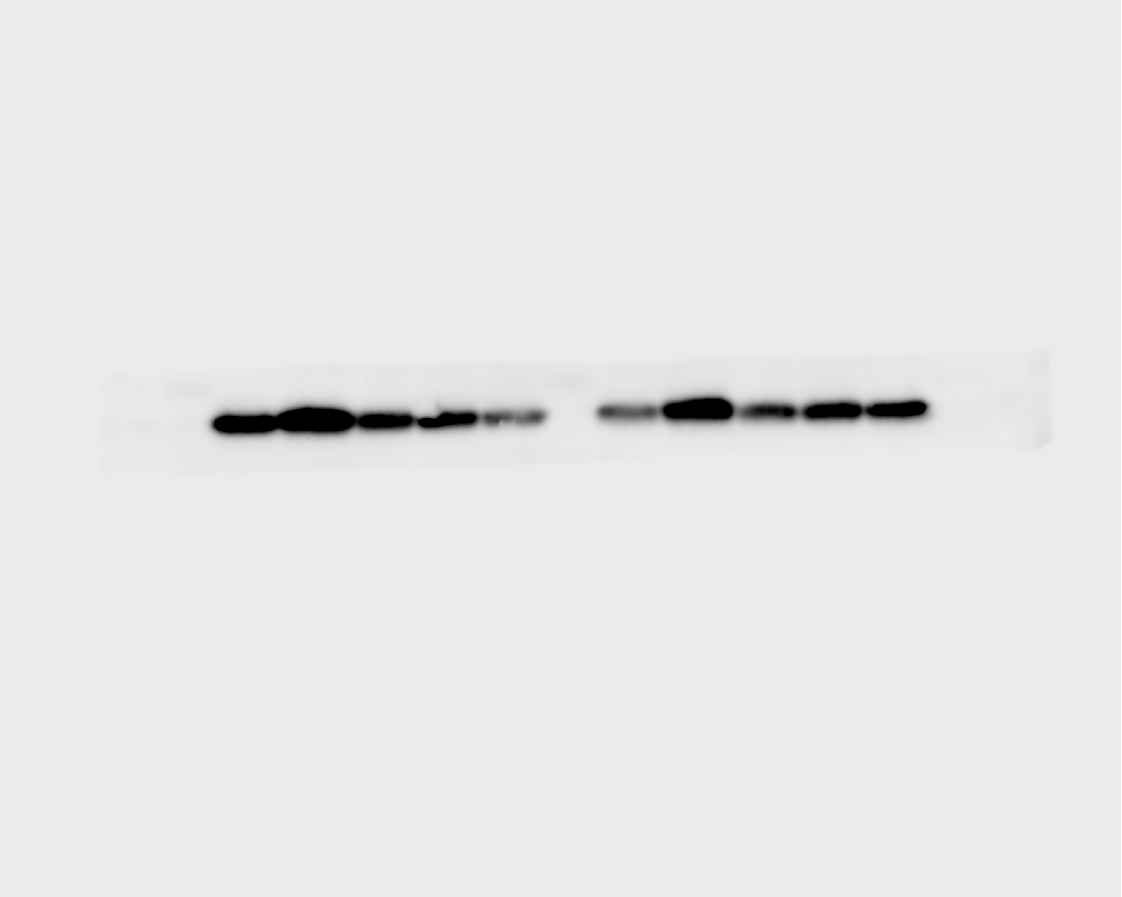


HMGB1 25KD


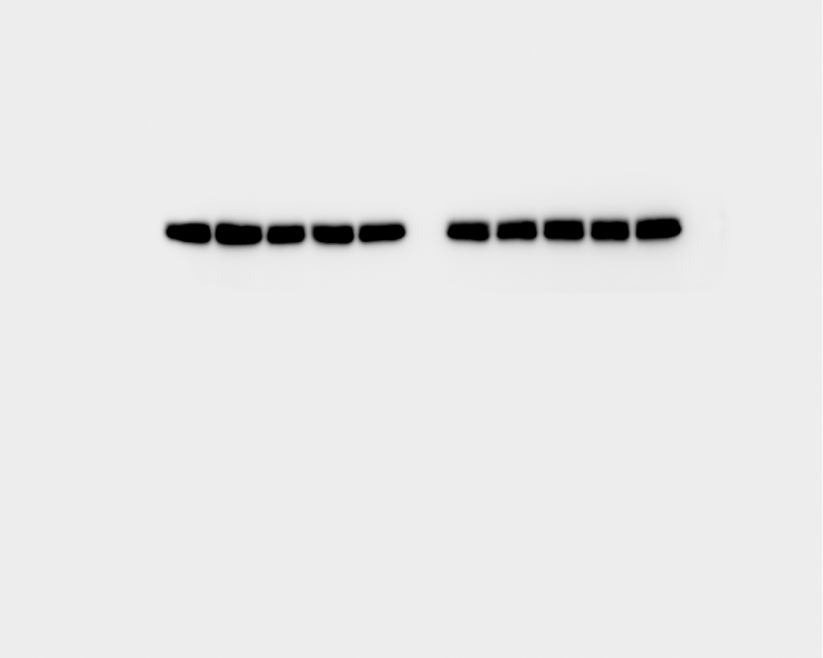


β-actin 43KD

20KD

Figure 5H

Lane 1: control; Lane 2: control; Lane 3: Vehicle+NMDA; Lane 4: Vehicle+NMDA; Lane 5: NMDA+MK-801; Lane 6: NMDA+MK-801; Lane 7: NMDA+AICAR; Lane 8: NMDA+AICAR; Lane 9: NMDA+LB-100; Lane 10: NMDA+LB-100; Lane 11: NMDA+GLY; Lane 12: NMDA+GLY


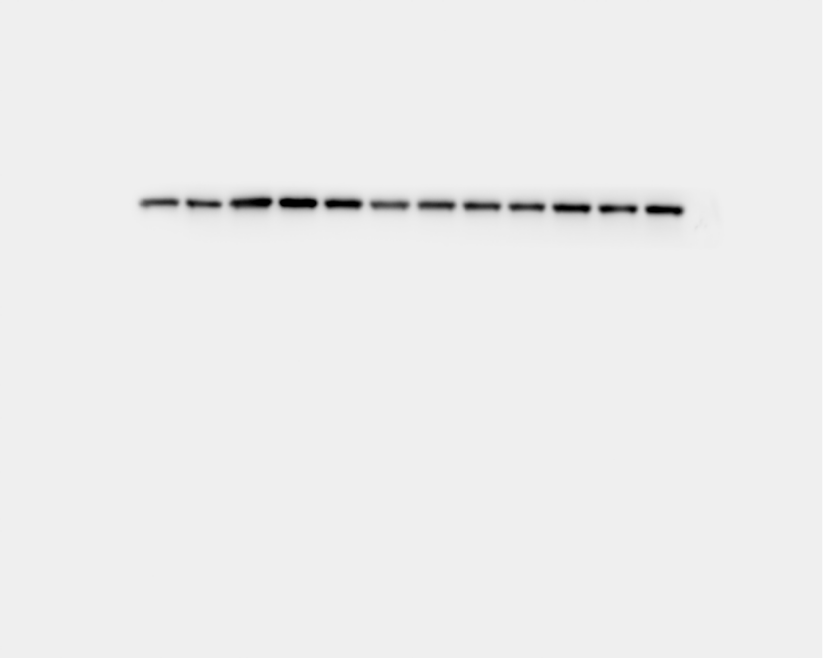


PTGS2 69KD


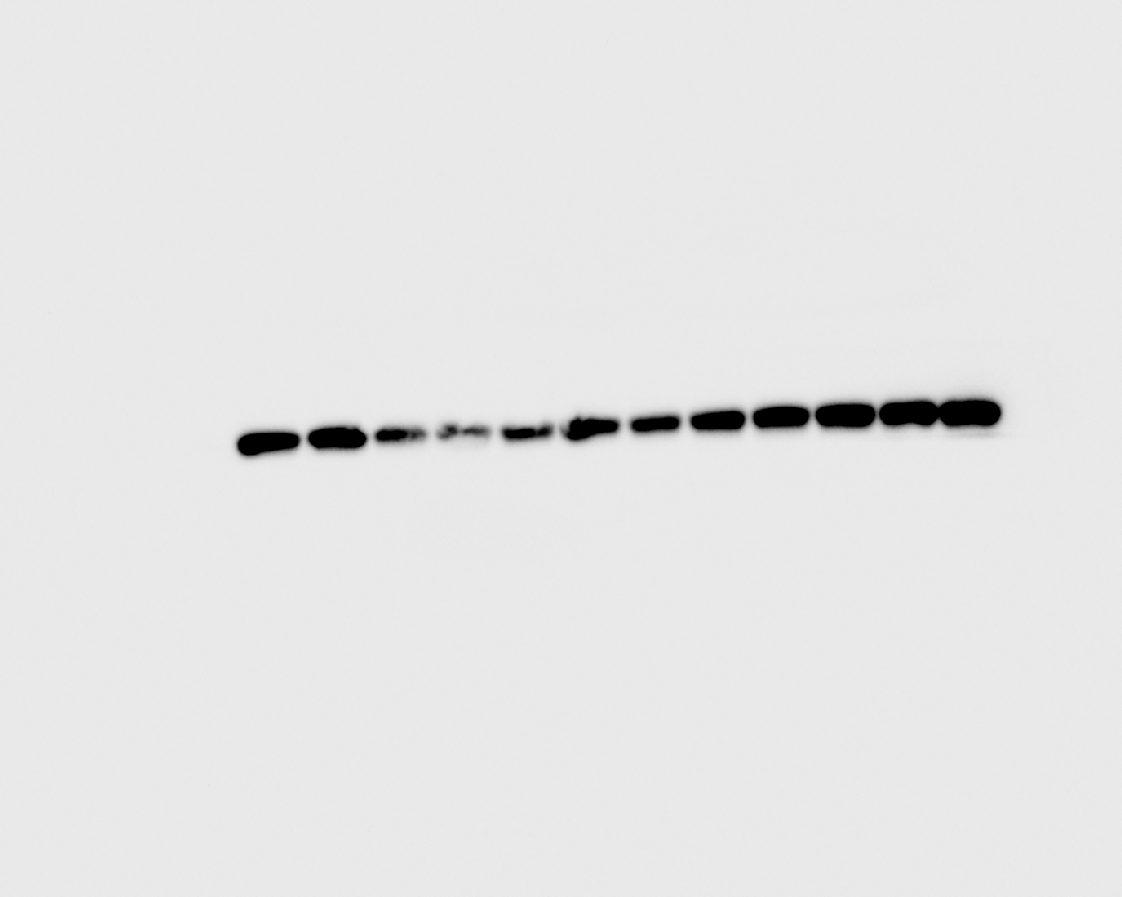


GPX4 20KD


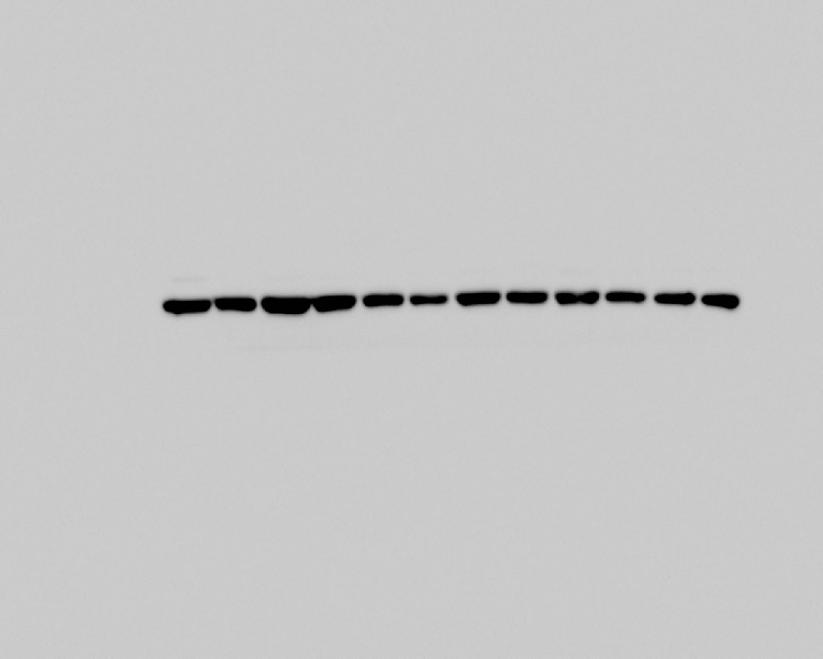


SLC7A11 55KD

20KD


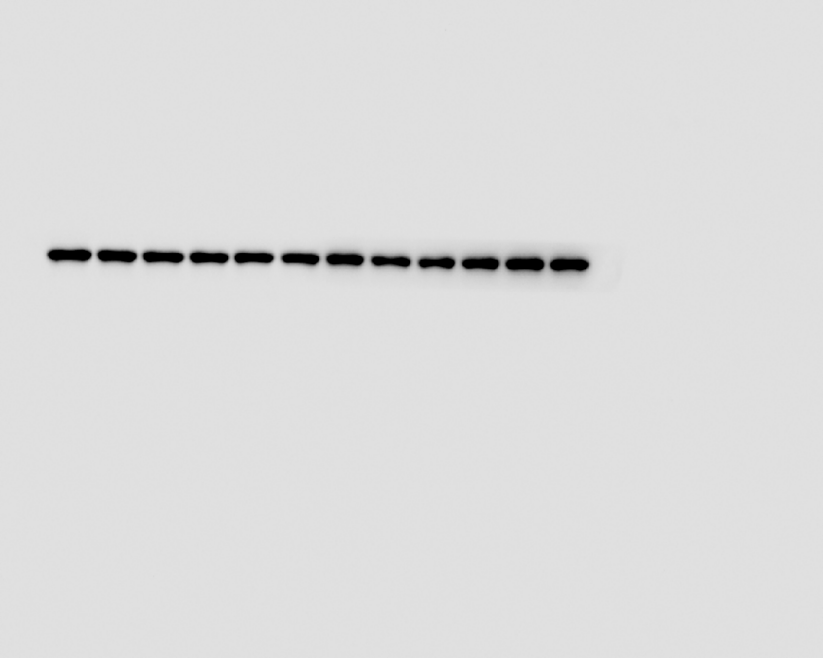


β-actin 43KD

20KD

Figure 5J

Figure 5J：PTGS2, GPX4, SLC7A11, β-actin

Lane 1: control; Lane 2: control; Lane 3: Vehicle + GLU; Lane 4: Vehicle + GLU; Lane 5: GLU + MK-801; Lane 6: GLU + MK-801; Lane 7: GLU + AICAR; Lane 8: GLU + AICAR; Lane 9: GLU + LB-100; Lane 10: GLU + LB-100; Lane 11: GLU + GLY; Lane 12: GLU + GLY


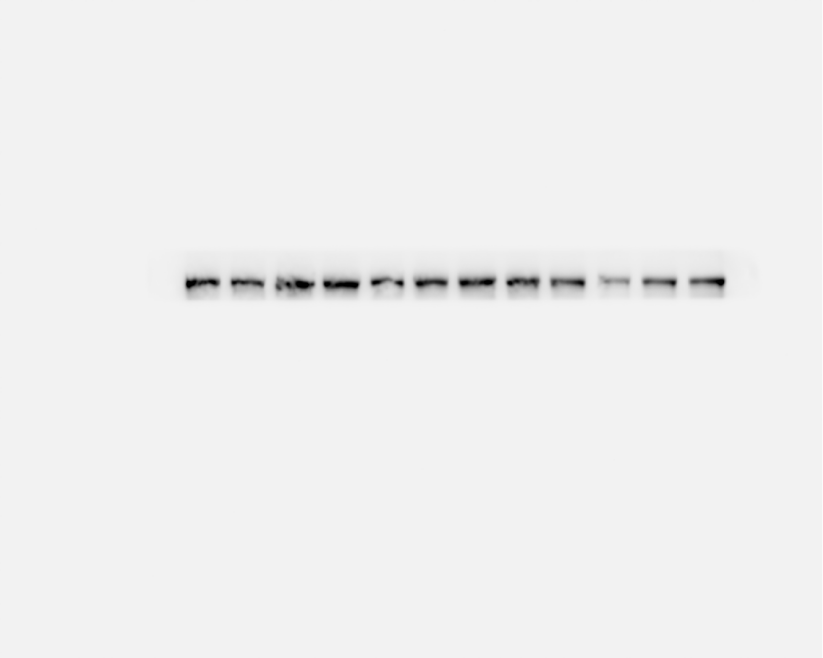


PTGS2 69KD


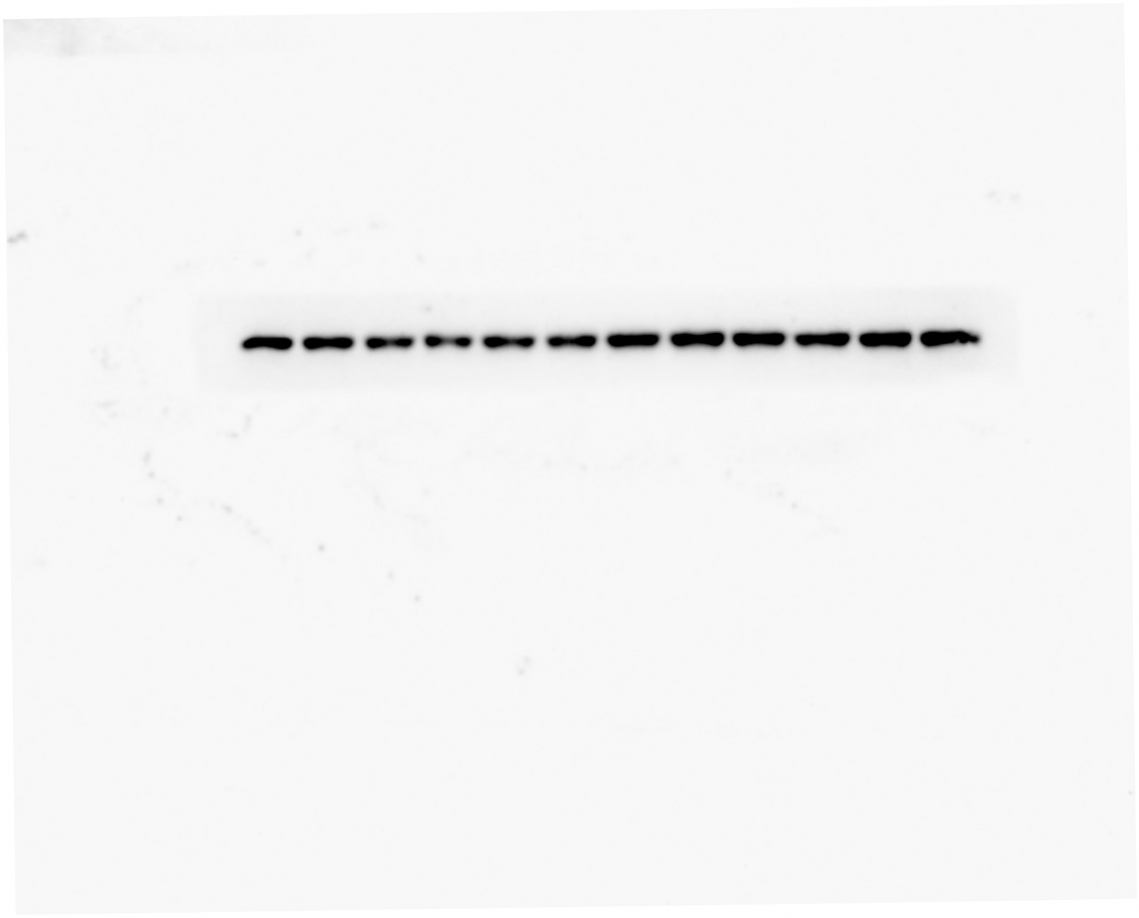


GPX4 20KD


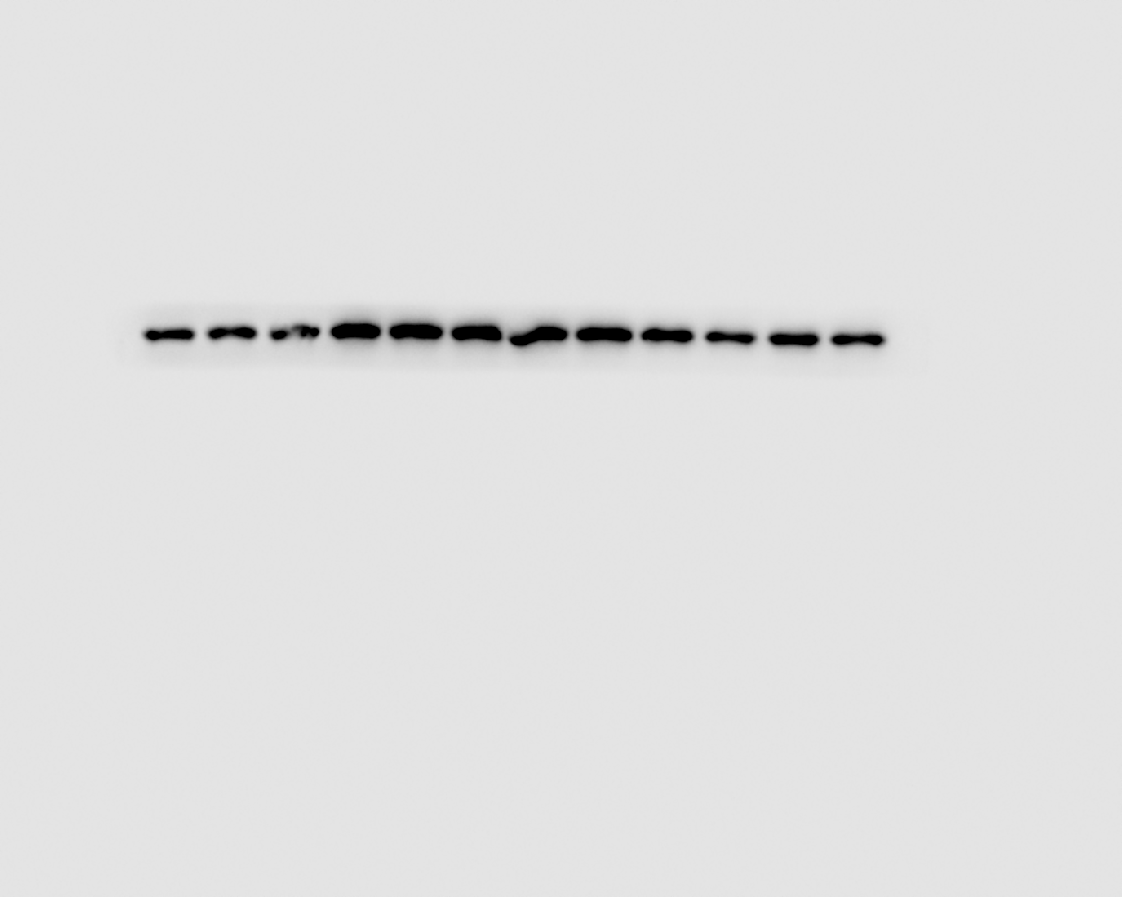


SLC7A11 55KD

20KD


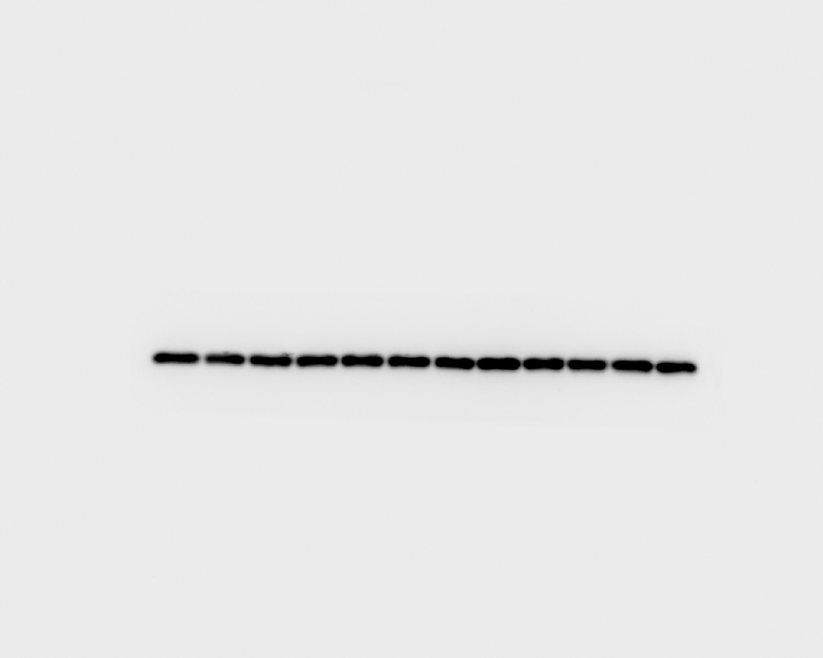


β-actin 43KD

20KD

Figure 6B

Lane 1: control; Lane 2: control; Lane 3: Vehicle+NMDA; Lane 4: Vehicle+NMDA; Lane 5: NMDA+MK-801; Lane 6: NMDA+MK-801; Lane 7: NMDA+AICAR; Lane 8: NMDA+AICAR; Lane 9: NMDA+LB-100; Lane 10: NMDA+LB-100; Lane 11: NMDA+GLYLane 12: NMDA+GLY


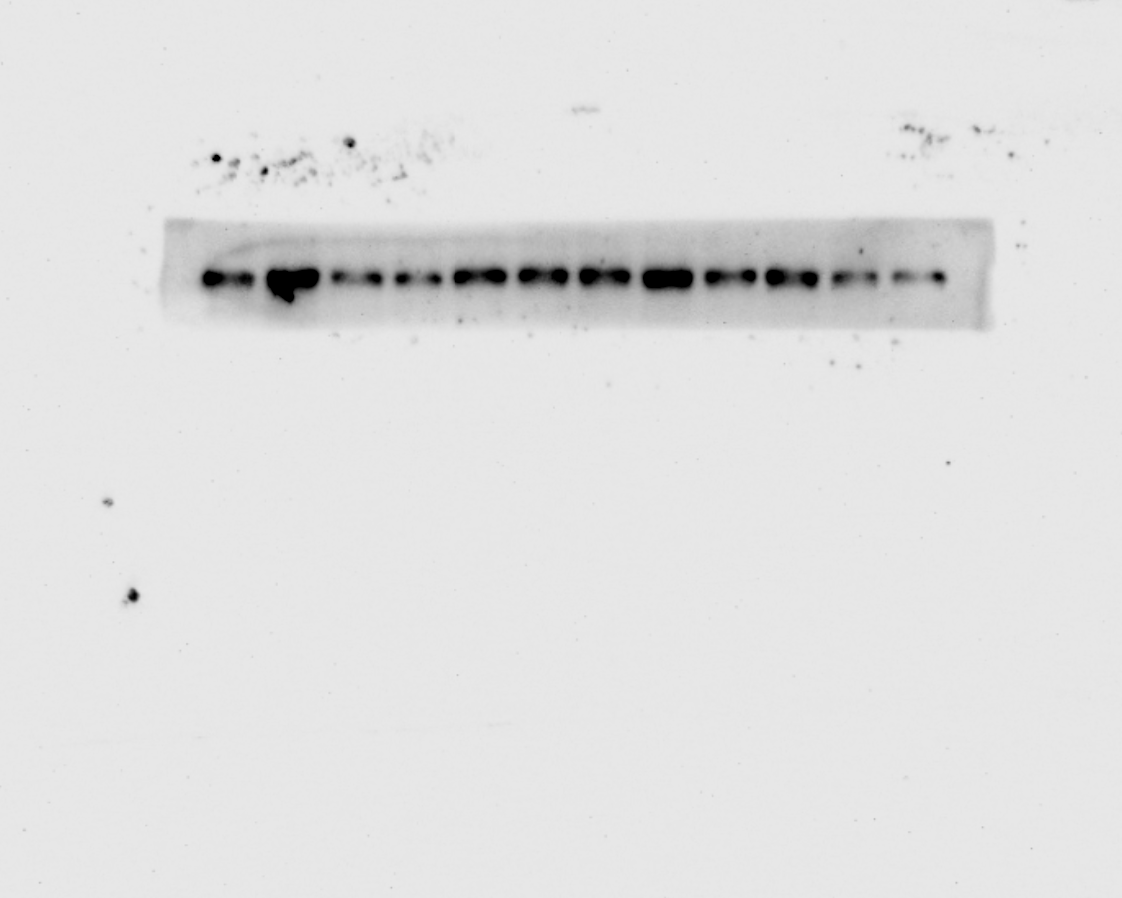

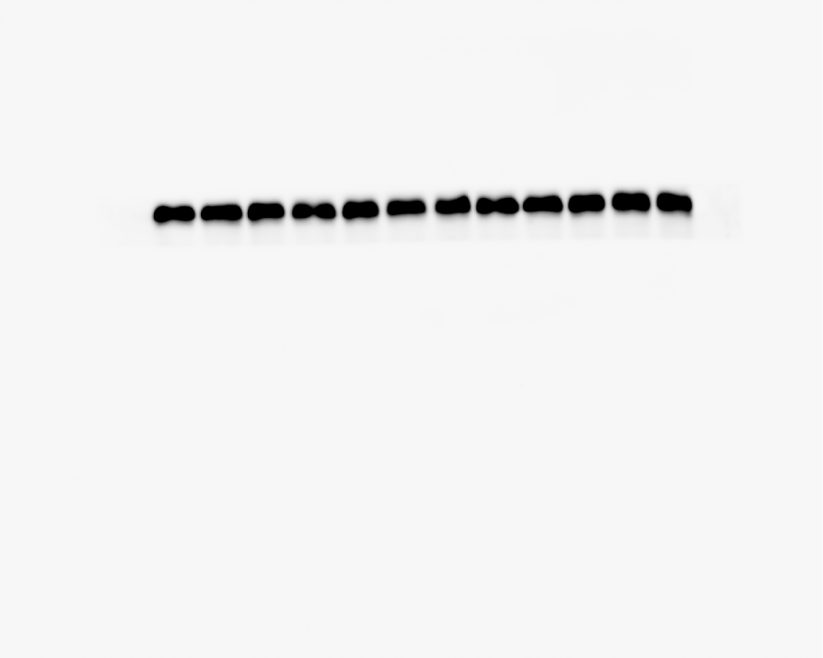


p-PP2A 36KD

t-PP2A 36KD


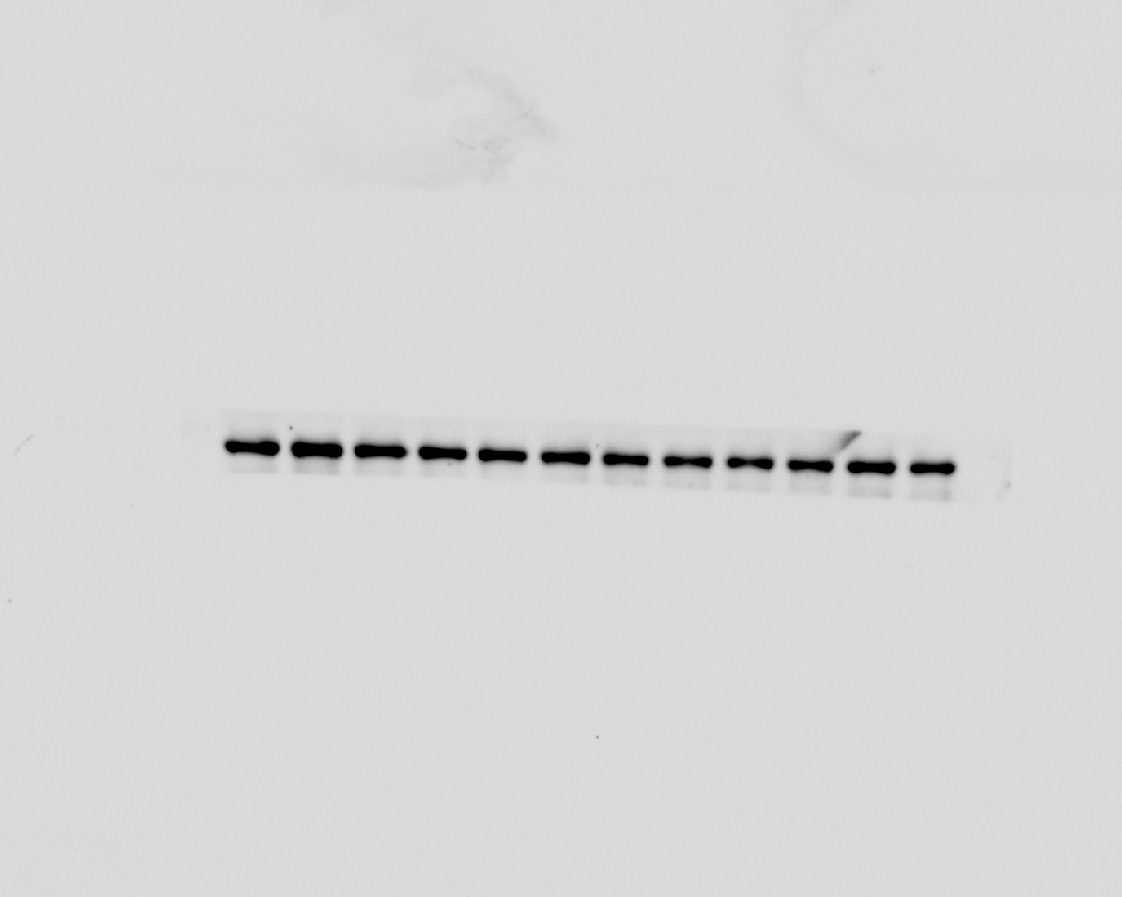


t-AMPK 62KD


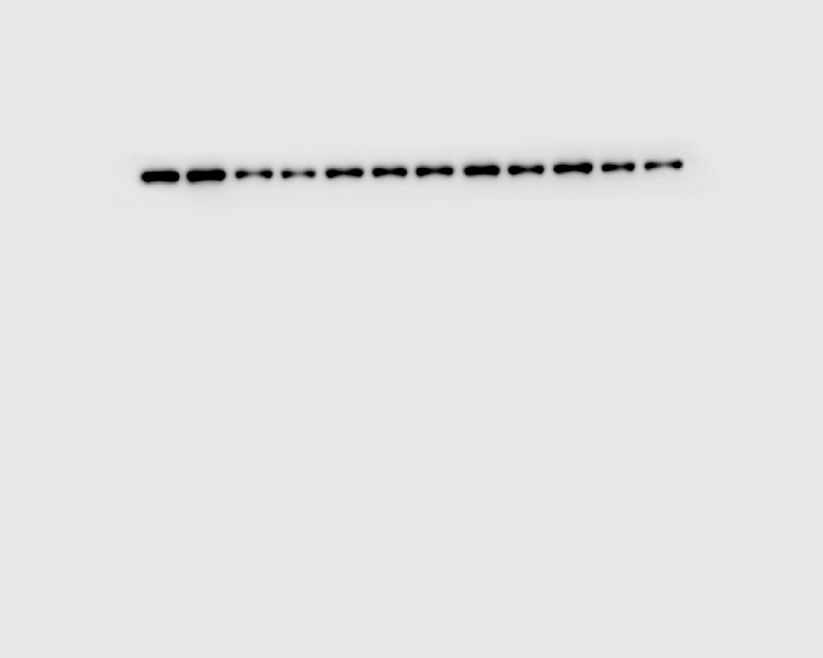

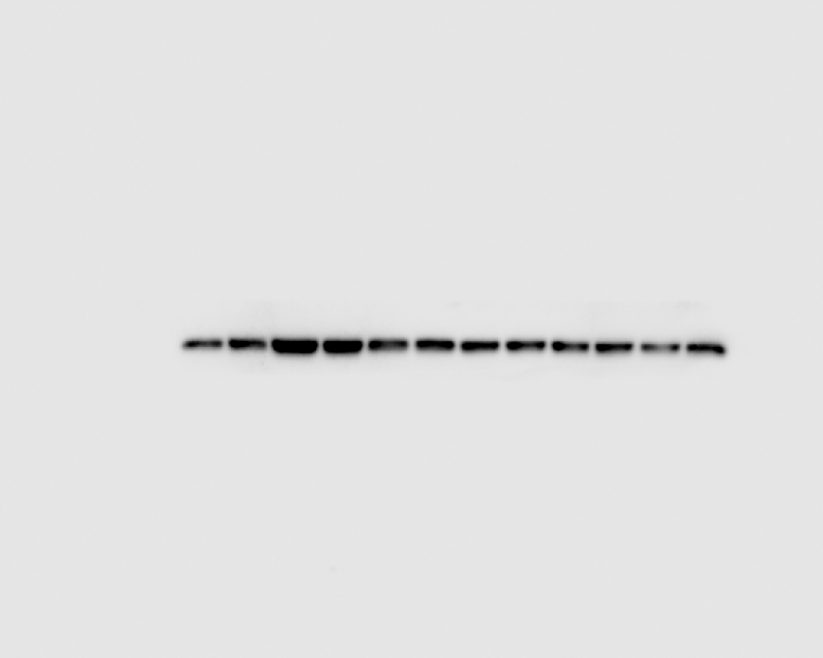


p-AMPK 62KD

HMGB1 25KD


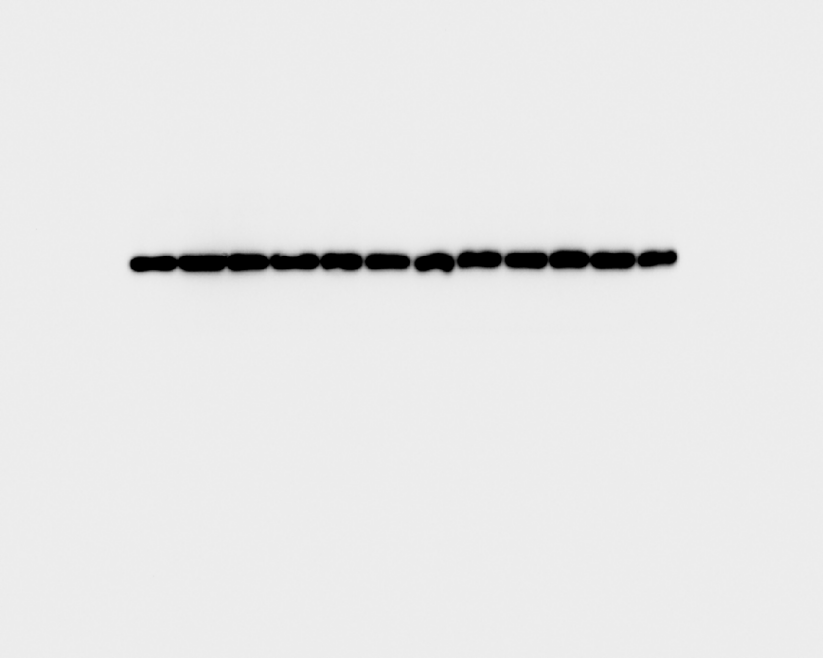


β-actin 43KD

20KD

Figure 6D: p-PP2A, t-PP2A, p-AMPK, t-AMPK, HMGB1, β-actin

Lane 1: control; Lane 2: control; Lane 3: Vehicle+GLU; Lane 4: Vehicle+GLU; Lane 5: GLU+MK-801; Lane 6: GLU+MK-801; Lane 7: GLU+AICA; Lane 8: GLU+AICAR; Lane 9: GLU+LB-100; Lane 10: GLU+LB-100; Lane 11: GLU+GLY; Lane 12: GLU+GLY


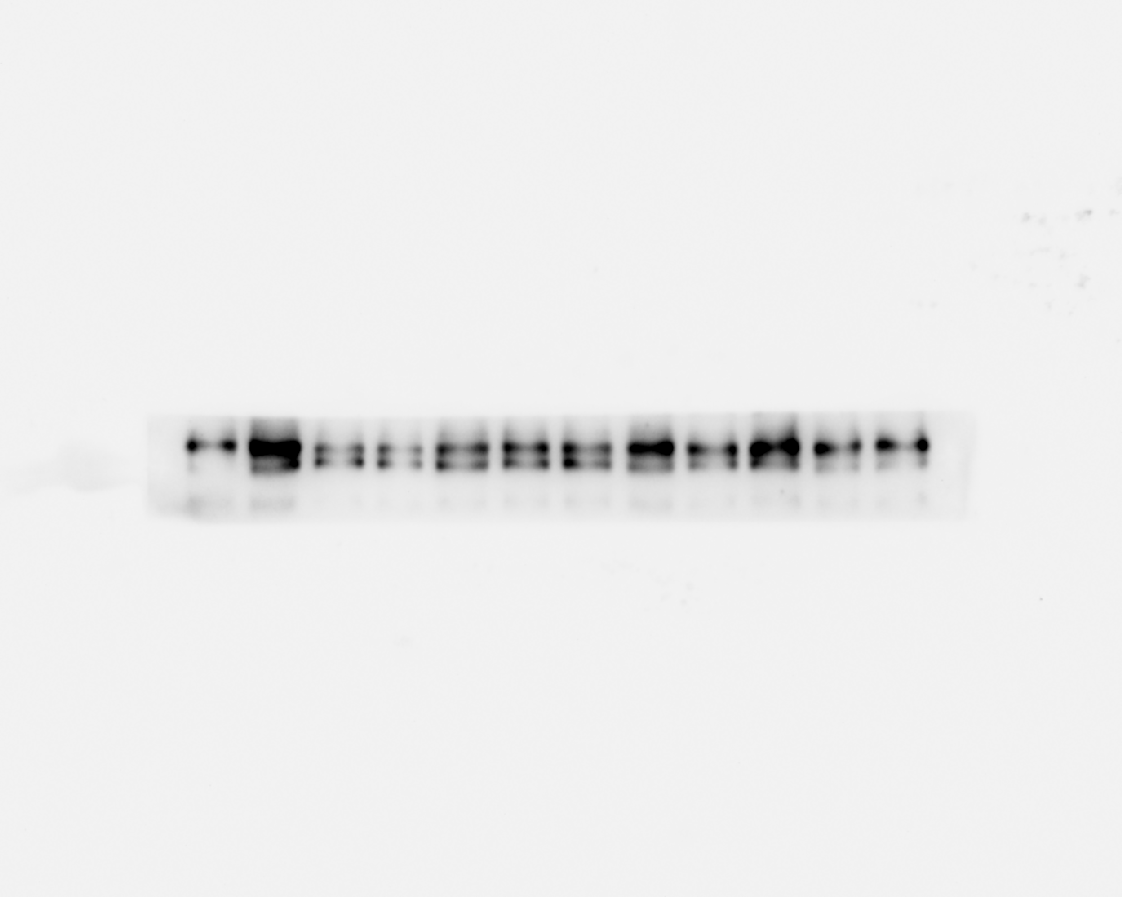


p-PP2A 36KD


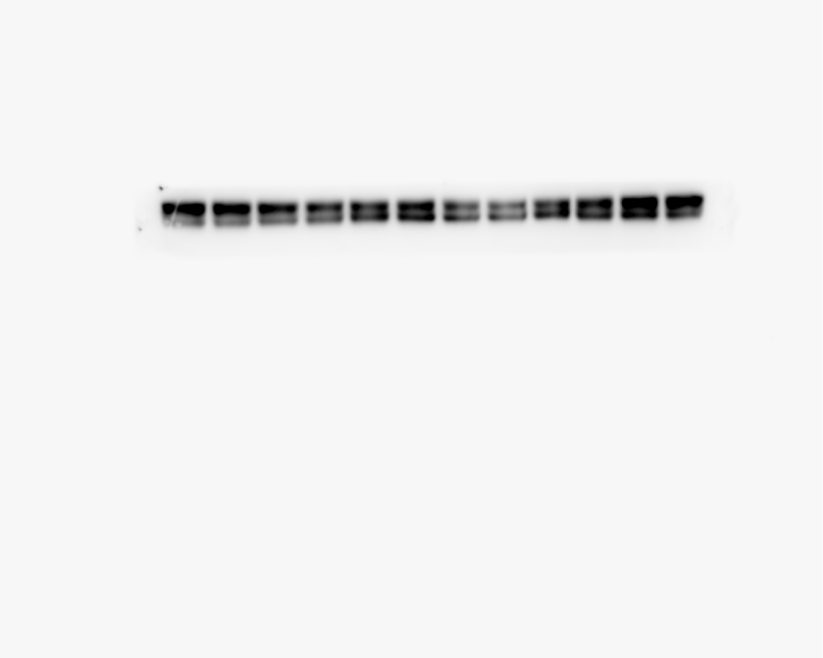


t-PP2A 36KD


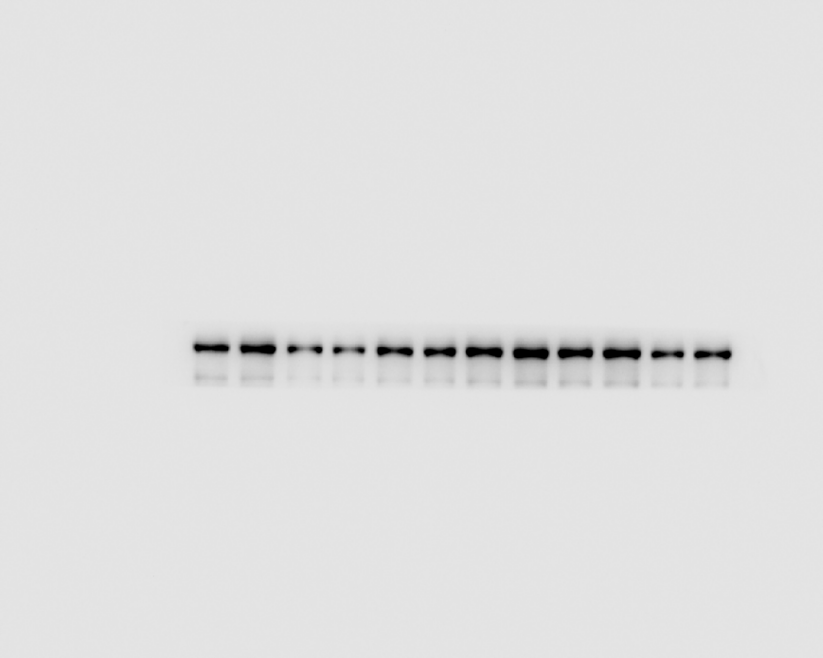


p-AMPK 62KD


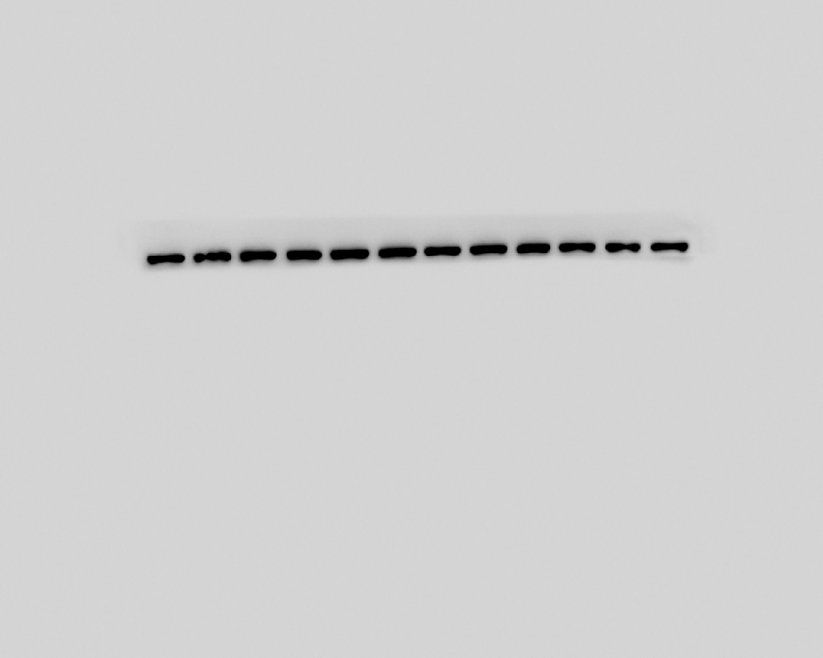


t-AMPK 62KD


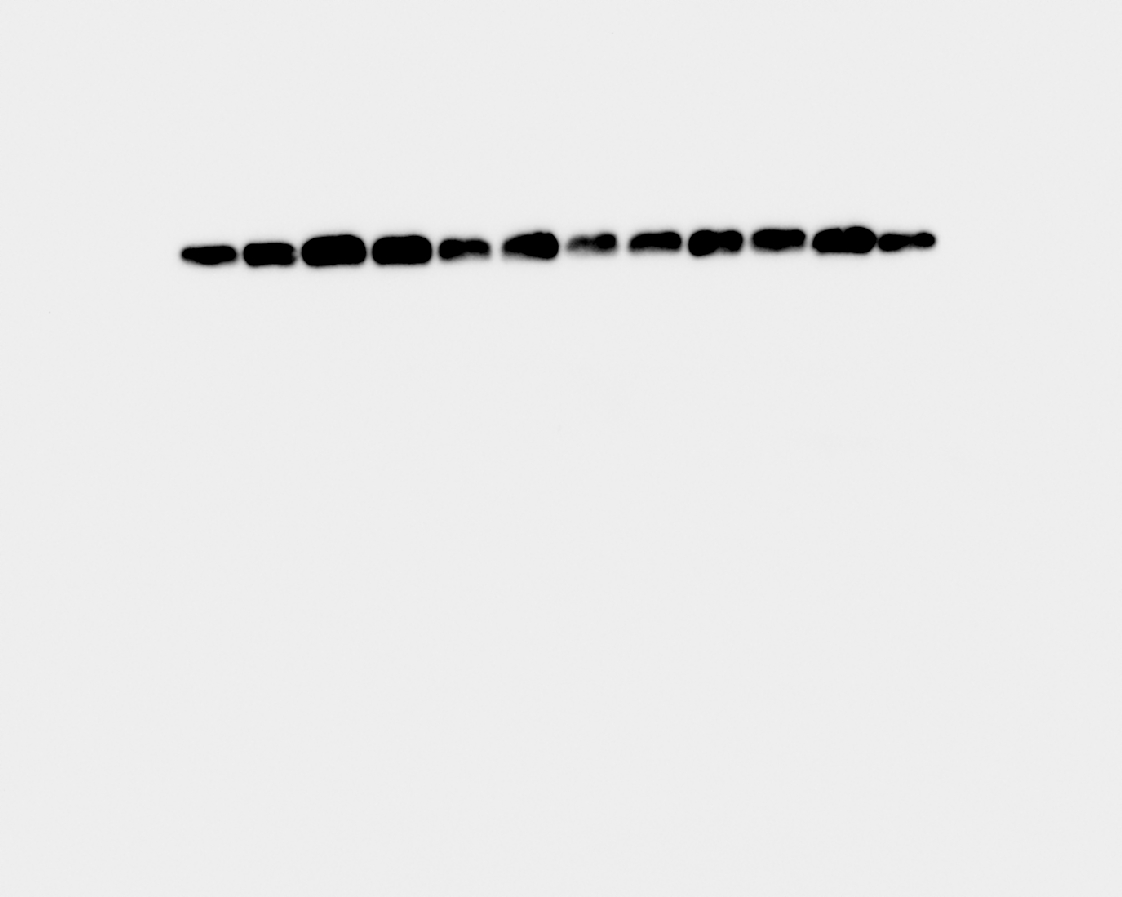


HMGB1 25KD


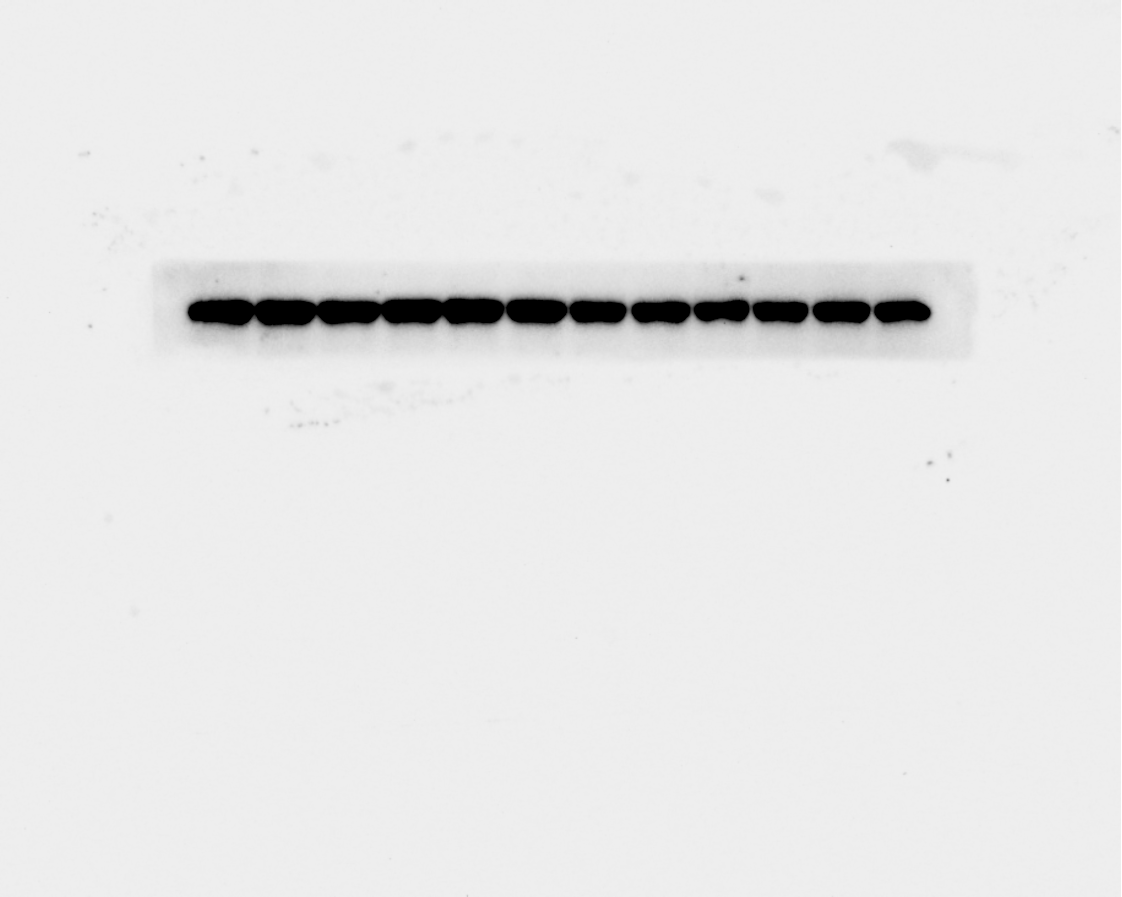


β-actin 43KD

20KD
